# Comic Zines as Tools for Chemistry Education and Engaging Students

**DOI:** 10.1021/acs.jchemed.4c00972

**Published:** 2024-10-12

**Authors:** Alexander B. Cook, Jan C. M. van Hest

**Affiliations:** Bio-Organic Chemistry, Institute for Complex Molecular Systems, Eindhoven University of Technology, 5600 MB Eindhoven, The Netherlands

**Keywords:** High School/Introductory Chemistry, First-Year Undergraduate/General, Communication, Student-Centered Learning, Collaborative/Cooperative Learning

## Abstract

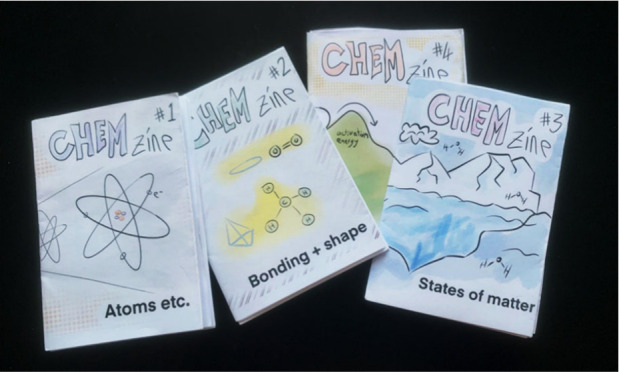

Use of solely language-based pedagogical tools in the teaching of chemistry can be limiting for such a practical and visual subject. In recent times, technology-based learning has become much more prevalent for engaging students. However, print based texts are still very important. Comics and comic zines have been shown to be attractive media forms for presenting information to students in an easily digestible form. Textbooks can be intimidating, and the presentation of chemistry syllabus with the addition of characters and often short narratives can help improve student engagement and scientific comprehension. Here, a series of self-made zines are investigated as tools for engaging and educating high school chemistry students (ages 16–18) in a visually appealing and accessible way. The pedagogic goals of the work are to increase motivation, increase student engagement, and investigate an innovative way of delivering the science curriculum for both students and teachers. Both content-based quizzes and student surveys indicate positive outcomes from zine supplemented study.

## 1 Introduction

Despite the importance of science and engineering in society, keeping students engaged in learning about science is a challenge.^1^ Attention is an important factor in the successful fulfillment of learning outcomes, and researchers have found that this attention on a learning task is more important than time spent on completing the task.^2^ Therefore, improving or directing student attention is a useful method for increasing achievement in schools and could lead to higher motivation for learning. In addition, young people’s motivation for, and knowledge of, scientific careers has been reported to be decreasing.^3,4^ Exposure to science, technology, engineering, and mathematics (STEM) content in school, among Gen Z pupils, can be correlated with postsecondary STEM interest. Pursuit of STEM university degrees and ultimately STEM careers are higher in students that are exposed to more STEM topics in high school.^3^ A recent report finds that even though 75% of Gen Z youth are interested in STEM occupations, only 29% list a STEM related job as their first-choice career.^4^ The development and use of new pedagogical methods are important for enhancing the interest and enjoyment of students in their science education and future careers.

Comics for science education and outreach are becoming more popular, with instructional cartoons for lab safety, and learning about acid/base theory, virology, and anatomy.^5−13^ However, zines as educational tools have been less well explored. In a simple definition, zines can be described as independent and self-made publications (by an individual or small group of people), often in a form of booklet or pamphlet, offering an inexpensive and simple route into the production and distribution of cultural and aesthetic works of interest.^14^ Compared to magazines; zines are often made by a single person with irregularity in frequency, while a magazine needs an editorial team and a fixed publishing schedule. Still, scientific magazines are also important for high school STEM education in both home and classroom settings.^15,16^ Current zine forms are thought to have originated in the 1920s when Science-Fiction fans would self-publish and share fan magazines with each other as a way to connect. Over time, this format transferred to comic books, rock/punk music centered zines, political zines, and many other topics not widely covered by mainstream publications. Their format can include text, be art-based, or be combinations of both.

There are a few published accounts of zines in education, mostly as student zine making activities, in science classes^17,18^ and humanities classes.^19−21^ There are two main distinctions for using zines in chemistry classes: Pre-existing or teacher-made zines and student made zines as a reflective exercise. Zine libraries can contain specific Science/Chemistry zines (for example see Small Science Collective^22^), however, the general lack of subject specific zines has possibly contributed to most educational research focusing on student made zine exercises. Zines as an educational tool to complement classroom learning or used course revision materials are underexplored.

In this article, a series of self-made zines are developed as a tool for enhancing high school chemistry learning (but also undergraduate students or other members of the public interested in the sciences). Zines broadly covering the syllabus of United Kingdom high school chemistry (atomic structure, bonding, states of matter, reaction equilibria and kinetics, organic chemistry, nucleic acids, and proteins) were distributed to students either via their teacher or through access to a Web site.^23^ The zines were designed to be concise summaries of sections of the syllabus with graphical, textual, and occasional character or metaphor use. The back pages were used to introduce a historic chemist important for that particular topic. On the basis of the strategic incorporation of visual and textual information, the hypothesis that CHEMzine would enhance student understanding of preuniversity chemistry and help them build intuition about the chemical and physical processes was tested. An online quiz covering aspects of the zines was used to assess educational outcomes from zine use. Questions were designed to measure information recall (IR), comprehension (C), and knowledge application (KA). The topics covered were not exclusive, and thus, the quiz could be answered without the zine activity/intervention. In order to account for this, there was a control group of students who answered the quiz questions but did not first look at the zines. Surveys of both students and teachers were also used to evaluate outcomes, such as student motivation and engagement. On the basis of these results and the survey outcomes, some suggestions are included to improve future classroom zine activities and hopefully stimulate further uptake of the comics and zines in science education.

## 2 Materials and Methods

Zines were designed to follow the final two years of the U.K. high school chemistry syllabus (AS/A level) divided into nine segments. The zines approximately follow the OCR syllabus (one of the U.K.’s five main examining bodies); however, the CHEMzine should be equally applicable to the other syllabi and even different countries. Each of the nine zines consists of six small pages of combined text and images, with the rear splash page showing a historic pioneer figure in that particular field of Chemistry and a small blurb explaining their research. The historic chemistry figures were chosen with equity and diversity in mind. Access to the CHEMzine is available through an open creative commons license at https://www.chemzine.com and also the Supporting Information (SI) of this article. They can be easily printed from a conventional printer and cut using scissors. Zine, quiz, and survey materials were distributed to teachers at 4 U.K. high schools through this web site and following that to students. The schools were state-funded schools, both selective and nonselective, of mixed ability students. Recommended use was as a course revision activity or general exam preparation. A zine takes approximately 5 min to read. Before accessing the zine materials (“pre-activity”) and after reading the zines (“post-activity”) a knowledge quiz (covering aspects from all zines and all topics) was conducted to assess learning and understanding outcomes. Performance in the quizzes was compared with a control group of students that followed the same course but without volunteering to use the zines. Students were assigned to either the control group or the experimental group on a self-selected basis.

### 2.1 Zine Preparation, Cutting, Folding

**Figure 1:**
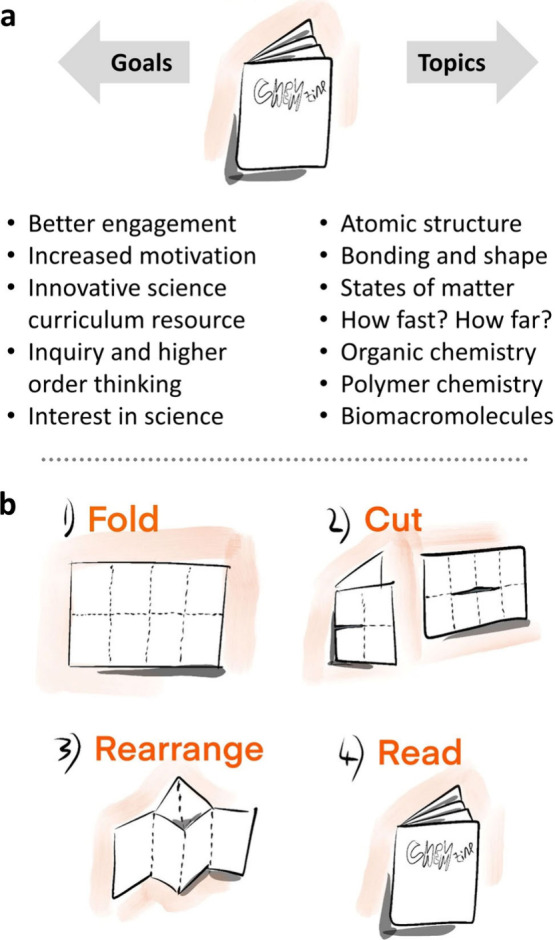
(a) Summary of the overall goals of this project as well as the general topics covered by the chemzine series, (b) cartoon depiction of the zine preparation instructions followed by students.

Zines were made following the nine topics and historical figures listed here: CHEMzine 1 - Atoms etc., Maria Goeppert-Mayer; CHEMzine 2 - Bonding and shape, Gilbert Lewis; CHEMzine 3 - States of matter, Johannes van der Waals; CHEMzine 4 - How fast? How far? Maud Menton; CHEMzine 5 - Organic chemistry, August Kekulé; CHEMzine 6 - More organic, Christopher Ingold; CHEMzine 7 - Polymer chemistry, Wallace Carothers; CHEMzine 8 - Information on life (DNA and RNA), Rosalind Franklin; CHEMzine 9 - Amino acids and proteins, Dorothy Hodgkin. One of the many positive aspects of using self-published zines as a resource is that they are completely open to use in different ways, with more or less words/artwork. For this work, the target word count in the zines was relatively low. Within the topics, various aspects were covered, including theory, historical facts, as well as equations and numerical concepts. There is flexibility in how teachers can use the zines; topics could be covered all in one session, or one by one after that topic is covered in class, for example. In this proof-of-concept study, teachers distributed the zines all together and, therefore, covered all topics in one session. CHEMzines were printed (two-sided) before the planned lesson, and students prepared their zines through folding and cutting along a defined section of the A4 paper (Figure 1).

### 2.2 Activity Analysis Methods

A total of 32 student responses were received for the zine cohort, and another 22 students for the control cohort. These students were A-Level chemistry students (age 16–18) and came from 4 different schools in the U.K. All were informed of their participation in this research project and provided their consent. To protect students’ confidentiality and security of their data, all data was collected anonymously. Practically, participating students carried out the preactivity quiz on an individual basis in the classroom setting before distribution of CHEMzine (designed with one 50 min class period in mind). Following an additional period of approximately 1 week, where the students also had access to the zines at home, students carried out the postactivity quiz and survey. Alternatively, instructors could save in-class time by assigning part or the entire activity to be completed outside of class time.

The assessed knowledge quiz was made up of 14 questions, with a selection of multiple choice and short open ended questions (SI). Questions were classified into assessment of information recall (IR), comprehension (C), and knowledge application (KA) randomly spread over the nine key topics of the course. An additional survey was distributed to gather information about student outcomes (SI). The survey included a number of demographic questions to start and then ten further questions based on a Likert scale format designed to assess comfort and motivation levels, as well as usefulness of comics based activities in science education. Students were asked to report their level of agreement or disagreement with the proposed statements with 5 options (strongly disagree, disagree, neutral, agree, strongly agree).

The quiz responses from all schools were anonymised and collected together for each question to give an average question score across participants. The average quiz scores of the question categories were compared within the student cohorts (zine use and control non zine use) using the two-tailed student *t* test. The average quiz score of the question categories was compared between zine and control cohorts also using the two-tailed student *t* test (Table S1). Cohen’s *d* value was used to calculate the effect size between the zine and control cohorts. For quizzes, significance was assigned with *p*-values in the following manner: * = *p*-val <0.05, ** = *p*-val <0.01, *** = *p*-val <0.001.

## 3 Results and Discussion

**Figure 2:**
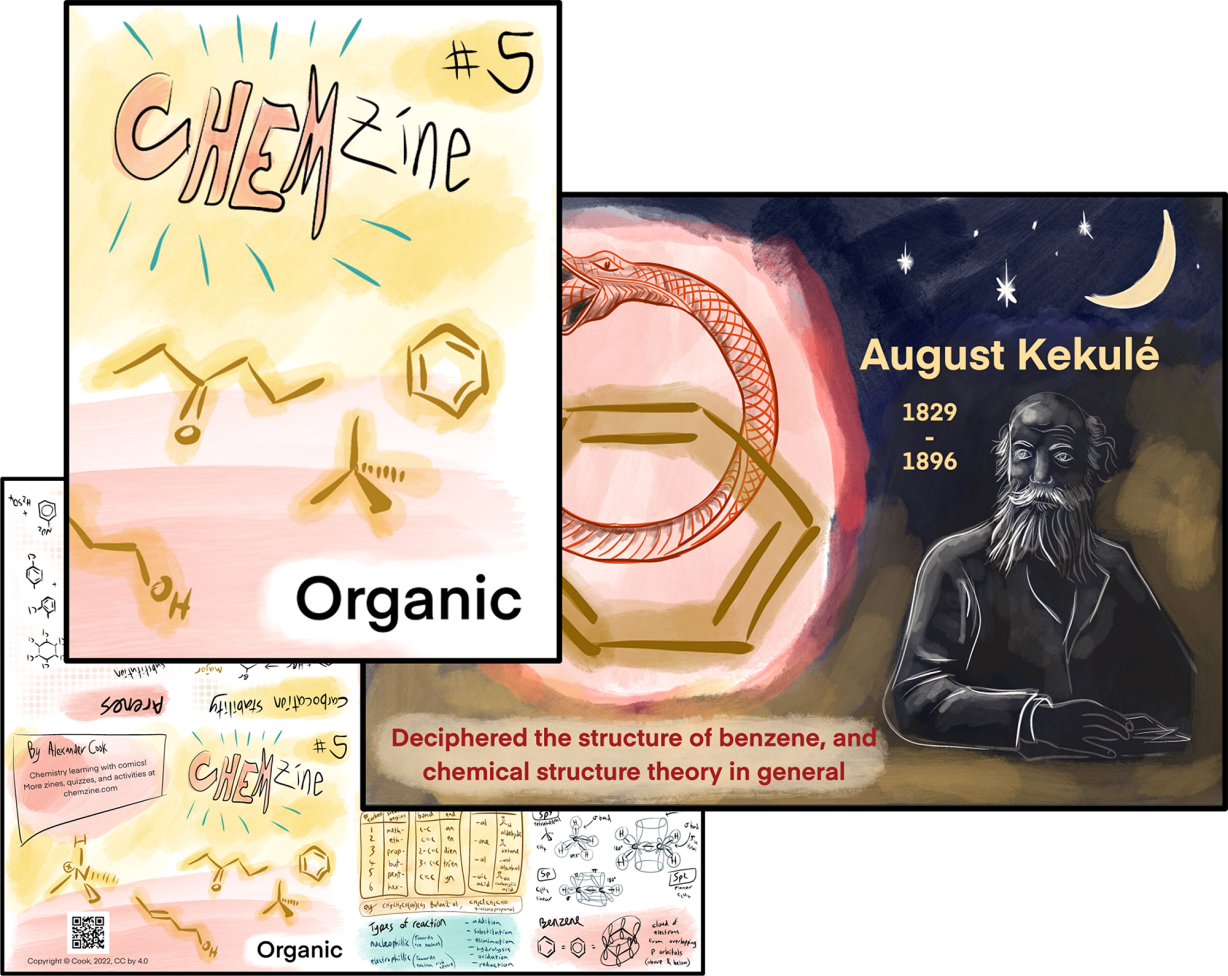
Example episode of CHEMzine, to be printed two-sided, which once folded, cut, and reassembled forms a concise and visually appealing summary of a course topic with an inspiring historical scientist featured on the back (when unfolded).

In general, CHEMzine was received positively by both students and instructors (an example of a zine is shown in Figure 2). Students were seen to be enthusiastically engaged in the assembling and reading of chemistry content in this colorful and tactile format. The inexpensiveness and self-published nature of the materials also allowed students to collect and share these mini textbooks between themselves, imparting a sense of ownership.

**Figure 3:**
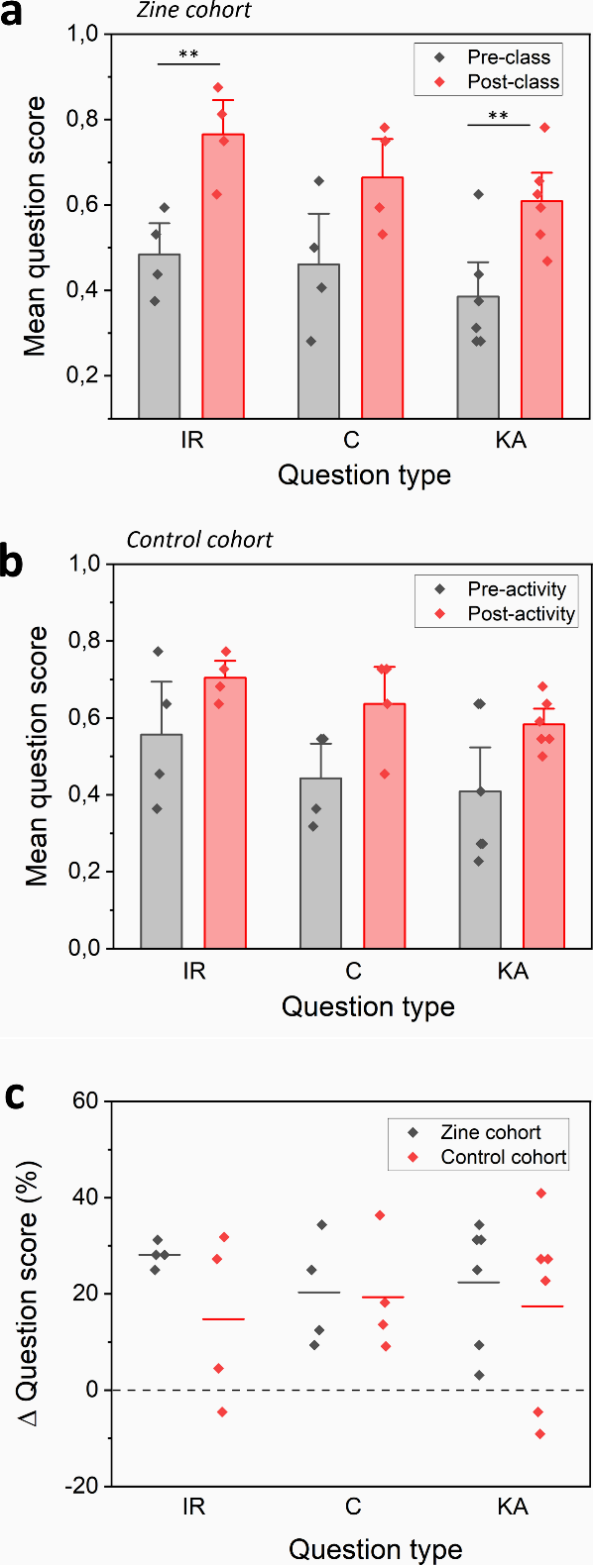
Quiz results for the chemistry content related questions from the zine cohort and the control cohort, each data point represents one question, (a) Zine cohort (*n* = 32 students) mean question scores (max = 1) for the information recall (IR), comprehension (C), and knowledge application (KA) categories, error bars represent standard deviation. (b) Control cohort (*n* = 22 students) mean question scores (max = 1) for the information recall (IR), comprehension (C), and knowledge application (KA) categories, error bars represent standard deviation. (c) Student performance improvement (expressed as%) before and after the zine activity. ** = *p*-val <0.01.

For each quiz question, scores of all students were collated and an average correct score calculated, expressed as a fraction with maximum = 1. In the preactivity quiz, students in the zine cohort had mean scores of 0.484 ± 0.097 and 0.461 ± 0.158 for the question categories information recall and comprehension, respectively (Figure 3a). While for the control cohort the mean scores were 0.557 ± 0.183 and 0.443 ± 0.120 for the same question categories (Figure 3b). These results were approximately in the same range and there was no significant difference between the two groups of students, as to be expected in a preactivity test, with both groups having previous exposure to the portions of the content. In the knowledge application question category, both groups scored slightly lower, 0.385 ± 0.132 and 0.409 ± 0.186 for the zine group and control group, respectively. This is possibly due to the limited previous exposure to this test format.

In the postactivity quiz, approximately 1 week following the classroom zine activity, both zine cohort students and control cohort students increased their scores in all question categories compared to their preactivity scores. The zine group’s scores increased by an average of 28%, 20%, and 22% for the categories IR, C, and KA, respectively. The increases in scores for the zine cohort were larger and statistically significant for IR and KA. While the control group’s scores increased by an average of 15%, 19%, and 17% for the categories IR, C, and KA respectively, these were not statistically significant increases compared to their preactivity results. We hypothesize that this score increase in the control group is due to repetition of the test with the same questions, as well as possible learning through normal classroom setting between sittings.

When comparing the postactivity quiz results from the two cohorts, the zine-use group had higher average question scores for all categories, with 0.758 ± 0.109, 0.664 ± 0.121, and 0.609 ± 0.108 for the question categories IR, C, and KA, respectively—compared to 0.705 ± 0.059, 0.636 ± 0.129, and 0.583 ± 0.067, for the non zine-use group. These increased values were statistically significant for the IR questions (Table S1). This suggests that the zine use may enhance the recall of specific chemistry knowledge and also problem-solving abilities in chemistry education. The information recall improvement could be due to the increased appeal of the zine format to visual and kinaesthetic learners.^24^ The increase in knowledge application question scores in the zine-use group can be attributed to the delivery of contextual and timely information alongside characters, questions, and simulations, which helps students observe connections among the content from a broad view, therefore deepening their understanding and ability to apply the knowledge to new questions.

**Figure 4:**
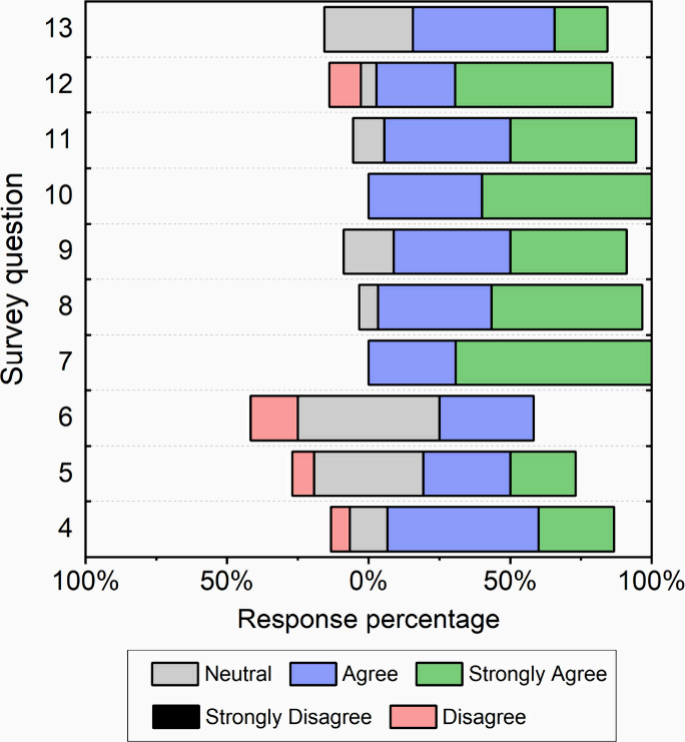
Survey results from postactivity collection (*n* = 16). The survey questions were presented on a five option Likert scale from strongly disagree to strongly agree.

Overall, feedback on the CHEMzine series was resoundingly positive. A survey to further evaluate both student and instructor reception of the zines was distributed. The results of the distributed survey questions are listed in Figure 4. CHEMzine survey feedback indicates that students who used the zine had increased understanding (Q4. Zine content helped me gain a better understanding of the course material), appreciation (Q7. My appreciation for the chemistry has improved as a result of reading these zines), interest (Q9. Zines can help me develop interest in chemistry), and enjoyment (Q11. I enjoyed using chemzines to study) of chemistry. High levels of agreement to these statements (80%–100% of respondents agreed or strongly agreed) indicate that students feel more engaged and enthusiastic about chemistry when presented in this format. The observation may correlate with the higher average question scores, i.e., higher engagement and enthusiasm, can lead to better understanding and evaluation performance.

Other noteworthy data from the user feedback survey include student responses to questions 5 (Use of zines helped me connect with other students) and 6 (Use of zines helped me connect with teacher), where results suggest zines increase student collaboration. In addition, student responses also indicate enthusiasm for the zine format for future learning (Q8. The format will be useful for exam/test revision; 93% agree or strongly agree; Q10. I would use zines to learn about new topics in the future, 100% agree or strongly agree; Q12. The zines could encourage me to study chemistry in the future, 83%). We received some extra comments from students, some of which are included here:

- “The zines were very helpful, likes summaries of the course material. I think they made the topics more understandable.”
- “The graphics in CHEMzine are produced nicely! It was very fun!”
- “I thought it was an interesting application for comics in chemistry. It was also interesting to learn about some chemistry history.”

## 4 Improvements

After the data and feedback on the use of CHEMzine in high school chemistry education were collected, some recommendations for teachers who are interested in employing similar tools in their course have been compiled. If zines were used as an instructor to distribute resources to students, as in this study, then one could consider distributing zines throughout the course as the topics arise, compared to all in one lesson as was done here. However, if one class is dedicated to the zines, then a revision session at the end of the course would be a good application. We also believe that other educational contexts could also be targeted. For example, zines could be used to help prepare and train students on particular laboratory techniques and instruments. Manga has been used to supplement laboratory safety messages; zines could likewise be beneficial in this regard.^6^ This project could further be combined with a STEM outreach activity; for example, many universities have school/family member open days; using zines in this context could be an engaging addition.

## 5 Conclusions

Comics, and zines in particular, are an easily created and shared, low tech communication medium through which anyone can impact their community. This article reports on the design and evaluation of a series of comic-inspired chemistry zines.^25−30^ CHEMzine is freely available online and has been used by high school students in the classroom setting (ages 16–18, from 4 U.K. schools). Through their unique combination of visual and verbal elements, this study has shown that zine use in high schools led to increased educational performance as well as self-reported enjoyment in chemistry learning. Reports have also linked increased enjoyment in creative and artistic pursuits to an increase in multidisciplinary scientific creativity.^31^

Topics covered in the zine series include atomic structure, bonding and shape, kinetics and thermodynamics, organic chemistry, polymer chemistry, and biomacromolecules. A scientific content based quiz was used to asses educational benefits of participating students, and results were compared to a control group of students who did not use the zines. Students with access to the zines had higher average question scores across the question categories of information recall, comprehension, and knowledge application. Average question scores were 0.758 ± 0.109, 0.664 ± 0.121, and 0.609 ± 0.108 for the question categories information recall (IR), comprehension (C), and knowledge application (KA), respectively—compared to 0.705 ± 0.059, 0.636 ± 0.129, and 0.583 ± 0.067, for the control group. The benefits, however, extend beyond test performance, as signs of increased individual creativity and self-direction were also observed, but less easily quantified. CHEMzine, and other class based zine reading or making activities, are not intended to replace current pedagogical techniques. However, comic zines and similar storytelling^32^ and gamification approaches^33−36^ may be useful additions to tools available for educators to engage students.

## Supporting Information Available

Assessment data table with two tailed student *t* test *p*-values and effect size Cohen’s *d*-values; assessment quiz questions; assessment survey questions; and printable PDF pages of CHEMzine’s for reproduction of this activity in classrooms/homes (PDF)
