# Supplementary material for: Comic Zines as Tools for Chemistry Education and Engaging Students
Source: J Chem Educ. 2024 Oct 12;102(2):929–34. doi: 10.1021/acs.jchemed.4c00972 (PMC11823410; doi:10.1021/acs.jchemed.4c00972)
Supplement: Supplementary file 1 — ed4c00972_si_001.pdf [file ed4c00972_si_001.pdf]

Supporting information

Comic zines as tools for chemistry education and engaging students

Alexander B Cook[a]*, Jan C M van Hest[ab]

[a]*Bio-Organic Chemistry, Institute for Complex Molecular Systems, Eindhoven University of Technology, Eindhoven, Netherlands*

[b]*Biomedical Engineering, Institute for Complex Molecular Systems, Eindhoven University of Technology, Eindhoven, Netherlands*

*Corresponding author: a.b.cook@tue.nl

Contents:

1. Assessment data table with two tailed student t-test p-values and effect size Cohen's d-values
2. Assessment quiz questions.
3. Assessment survey questions.
4. Printable PDF pages of CHEMzine's for reproduction of this activity in classrooms/homes.

	Zine cohort (n=32)							
	Pre-activity				Post-activity			
	Ave.	SD	P value	Cohen'sd (effect size)	Ave.	SD	P value	Cohen's d (effect size)
Information recall (4 Q.)	0,484	0,097	0,062	-0,49	0,758	0,109	**0,043 (*)**	0,61
Comprehension (4 Q.)	0,461	0,158	0,653	0,13	0,664	0,121	0,420	0,22
Knowledge application (6 Q.)	0,385	0,132	0,581	-0,15	0,609	0,108	0,321	0,29

	Control cohort (n=22)			
	Pre-activity		Post-activity	
	Ave.	SD	Ave.	SD
Information recall (4 Q.)	0,557	0,183	0,705	0,059
Comprehension (4 Q.)	0,443	0,120	0,636	0,129
Knowledge application (6 Q.)	0,409	0,186	0,583	0,067

Table S1. *Summary table of activity quiz analysis statistics, mean and standard deviation, of quiz scores out of 1. Qustions were bracketed into categories of information recall (IR), comprehension (C), and knowledge application (KA). P-values (two tailed student t-test) and effect size (Cohen's d) were obtained from comparing the zine cohort values to corresponding pre or post activity values from the control cohort, * = p-val < 0.05.*

CHEMzine quiz

Chemistry content questions

1. What particles make up the nucleus of an atom? — 1 point

2. What is an isotope? — 1 point

3. The below atom has how many protons and neutrons? — 1 point

$$^{234}_{90}Th$$

Mark only one oval.

- ( ) 90 protons and 144 neutrons
- ( ) 234 protons and 90 neutrons
- ( ) 0 protons and 90 neutrons

4. Place the molecules in order of decreasing bond angles. — 1 point

SF_6 BeF_2 F_2O SO_3

5. Identify X, Y, and Z. — 3 points

	Melting point	Electrical conductivity
X	1683 K	Very poor
Y	4900 K	Very high
Z	386 K	None

Mark only one oval per row.

	Silicon	Sulfur	Graphene
**X**	( )	( )	( )
**Y**	( )	( )	( )
**Z**	( )	( )	( )

6. This is the Boltzman distribution showing activation energy for a reaction. When a catalyst is used, which is the correct new activation energy? — 1 point

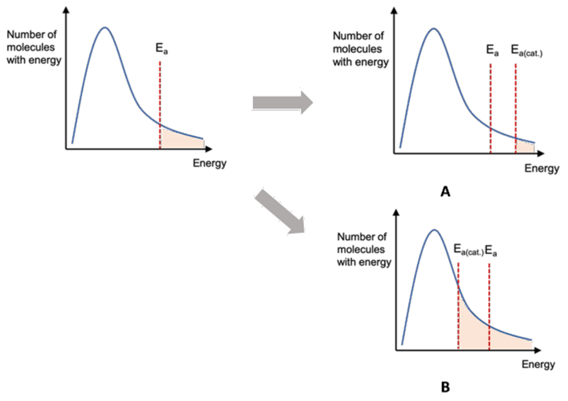

Mark only one oval.

- ( ) A
- ( ) B

7. Which of the following molecules is 3,3-dimethylpentan-2,4-dione? 1 point

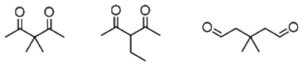

A B C

Mark only one oval.

- A
- B
- C

8. Name the following compound. 1 point

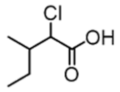

Mark only one oval.

- 2-chloro-3-methylpentanoic acid
- 2-ethyl-3-chlorobutanoic acid
- 2-chlorohexanoic acid

9. Name the following class of reaction: 1 point

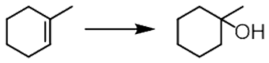

Mark only one oval.

- Nucleophilic substitution
- Electrophilic addition
- Reduction

10. Name the following class of reaction: 1 point

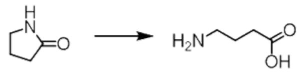

Mark only one oval.

- Hydrolysis
- Electrophilic addition
- Oxidation

11. What reducing agent would you use for the following reaction? 1 point

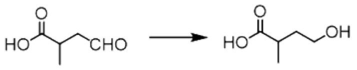

Mark only one oval.

- LiAlH4
- NaBH4

12. What is the structure of the condensation polymer made from the following monomers? 1 point

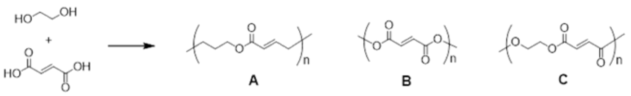

Mark only one oval.

- A
- B
- C

13. The sequence of amino acids in a protein is called what? 1 point

Mark only one oval.

- Primary structure
- Secondary structure
- Tertiary structure

14. The two strands in a DNA double helix are held together by what forces? 1 point

Mark only one oval.

- H-Bonding
- Electrostatic forces
- Gravity

This content is neither created nor endorsed by Google.

Google Forms

CHEMzine feedback

Please fill out to help with our evaluation

1. Which school are you from?

2. Are you a student or teacher?

   Mark only one oval.

   - Student
   - Teacher

3. How old are you?

4. Zine content helped me gain a better understanding of the course material.

   Mark only one oval.

   - Strongly agree
   - Agree
   - Neutral
   - Disagree
   - Strongly disagree

5. Use of zines helped me connect with other students.

   Mark only one oval.

   - Strongly agree
   - Agree
   - Neutral
   - Disagree
   - Strongly disagree

6. Use of zines helped me connect with teacher.

   Mark only one oval.

   - Strongly Agree
   - Agree
   - Neutral
   - Disagree
   - Strongly disagree

7. My appreciation for the chemistry has improved as a result of reading these zines.

   Mark only one oval.

   - Strongly Agree
   - Agree
   - Neutral
   - Disagree
   - Strongly disagree

8. The format will be useful for exam/test revision.

   Mark only one oval.

   - Strongly Agree
   - Agree
   - Neutral
   - Disagree
   - Strongly disagree

9. Zines can help me develop interest in chemistry.

Mark only one oval.

- Strongly agree
- Agree
- Neutral
- Disagree
- Strongly disagree

10. I would use zines to learn about new topics in the future.

Mark only one oval.

- Strongly Agree
- Agree
- Neutral
- Disagree
- Strongly disagree

11. I enjoyed using chemzines to study.

Mark only one oval.

- Strongly Agree
- Agree
- Neutral
- Disagree
- Strongly disagree

12. The zines could encourage me to study chemistry in the future.

Mark only one oval.

- Strongly agree
- Agree
- Neutral
- Disagree
- Strongly disagree

13. I would like to make my own zines for studying in the future.

Mark only one oval.

- Strongly agree
- Agree
- Neural
- Disagree
- Strongly disagree

This content is neither created nor endorsed by Google.

Google Forms

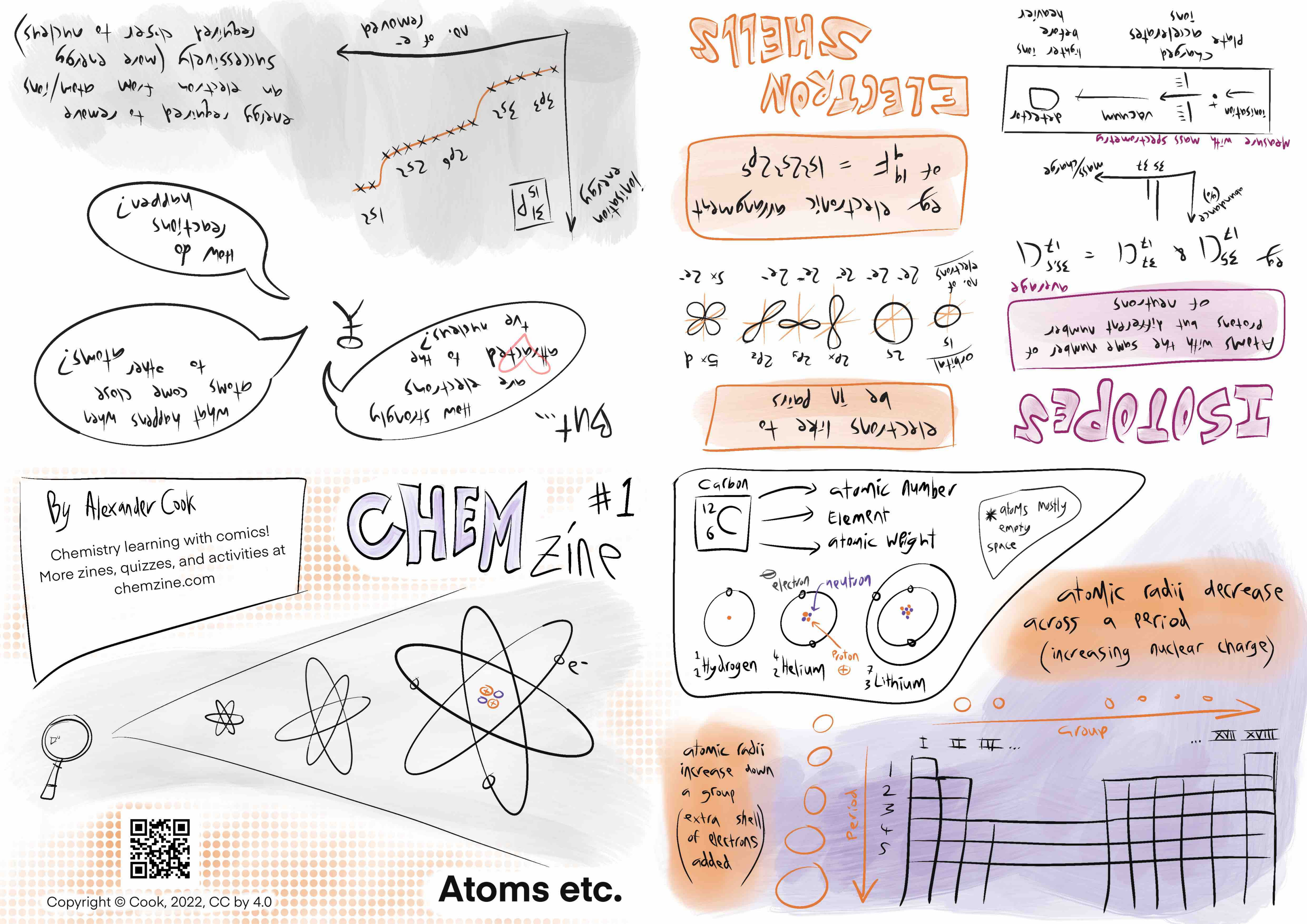
By Alexander Cook
Chemistry learning with comics!
More zines, quizzes, and activities at chemzine.com
CHEM zine #1
e-
Atoms etc.
Copyright © Cook, 2022, CC by 4.0
Carbon
12 6 C
atomic number
Element
atomic weight
* atoms mostly empty space
electron
neutron
proton
1 1 Hydrogen
4 2 Helium
7 3 Lithium
atomic radii decrease across a period (increasing nuclear charge)
Group
I II III ... XVII XVIII
Period
1 2 3 4 5
atomic radii increase down a group (extra shell of electrons added)
What happens when atoms come close to other atoms?
How do reactions happen?
But...
How strongly are electrons attracted to the +ve nucleus?
energy required to remove an electron from atom/ions successively (more energy required closer to nucleus)
ionisation energy
no. of e- removed
1s2 2s2 2p6 3s2 3p3
31 15 P
ELECTRON SHELLS
eg electronic arrangement of 19 9 F = 1s2 2s2 2p5
electrons like to be in pairs
orbital 1s 2s 2px 2py 2pz 5x d
no. of electrons 2e- 2e- 2e- 2e- 2e- 5x 2e-
ISOTOPES
Atoms with the same number of protons but different number of neutrons
eg 35 17 Cl & 37 17 Cl = 35.5 17 Cl
average
abundance (%)
mass/charge
35 37
Measure with mass spectrometry
ionisation
vacuum
detector
charged plate accelerates ions
lighter ions before heavier

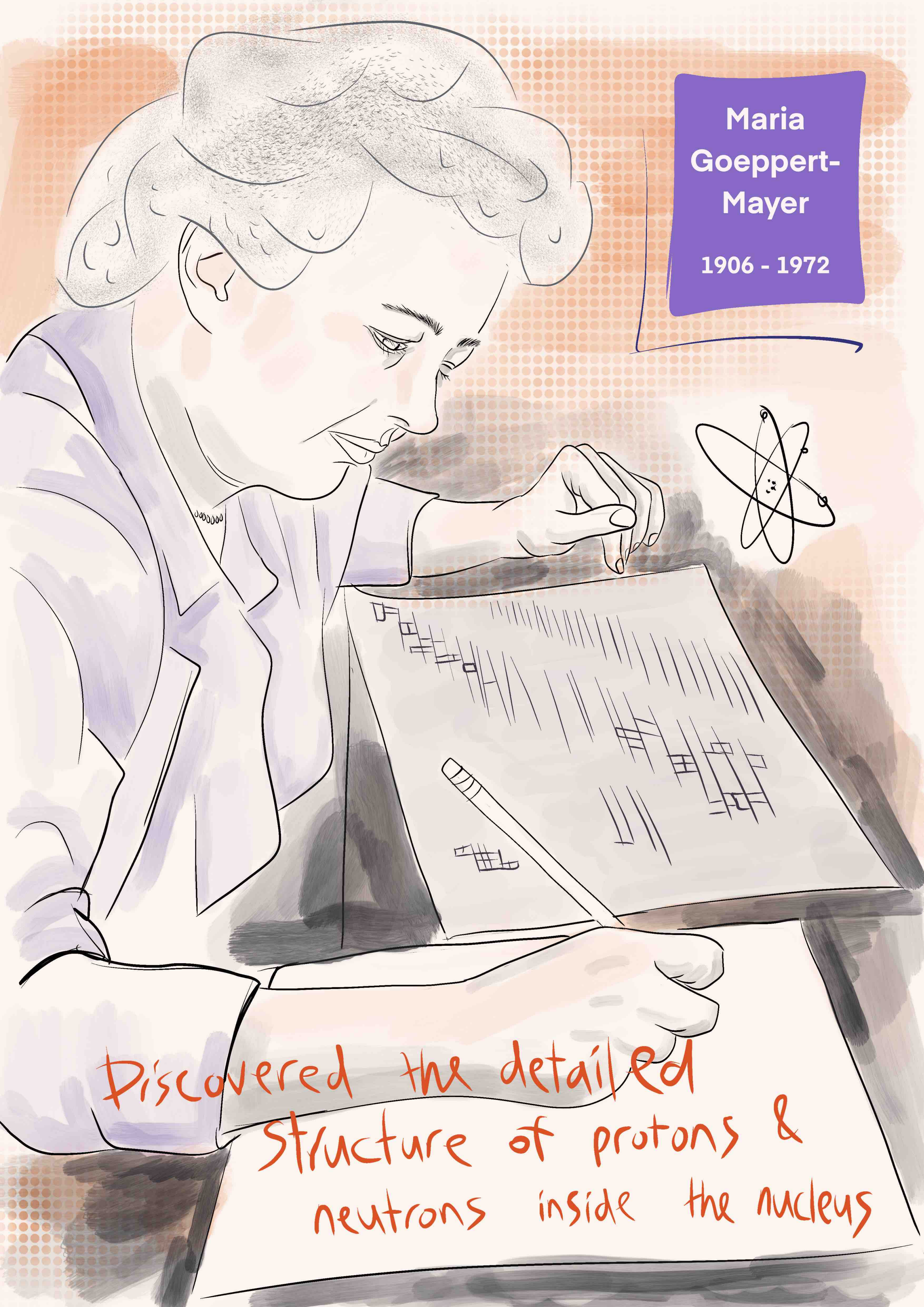
Maria Goeppert-Mayer
1906 - 1972
Discovered the detailed Structure of protons & neutrons inside the nucleus

CHEM zine #2

By Alexander Cook

Chemistry learning with comics!
More zines, quizzes, and activities at
chemzine.com

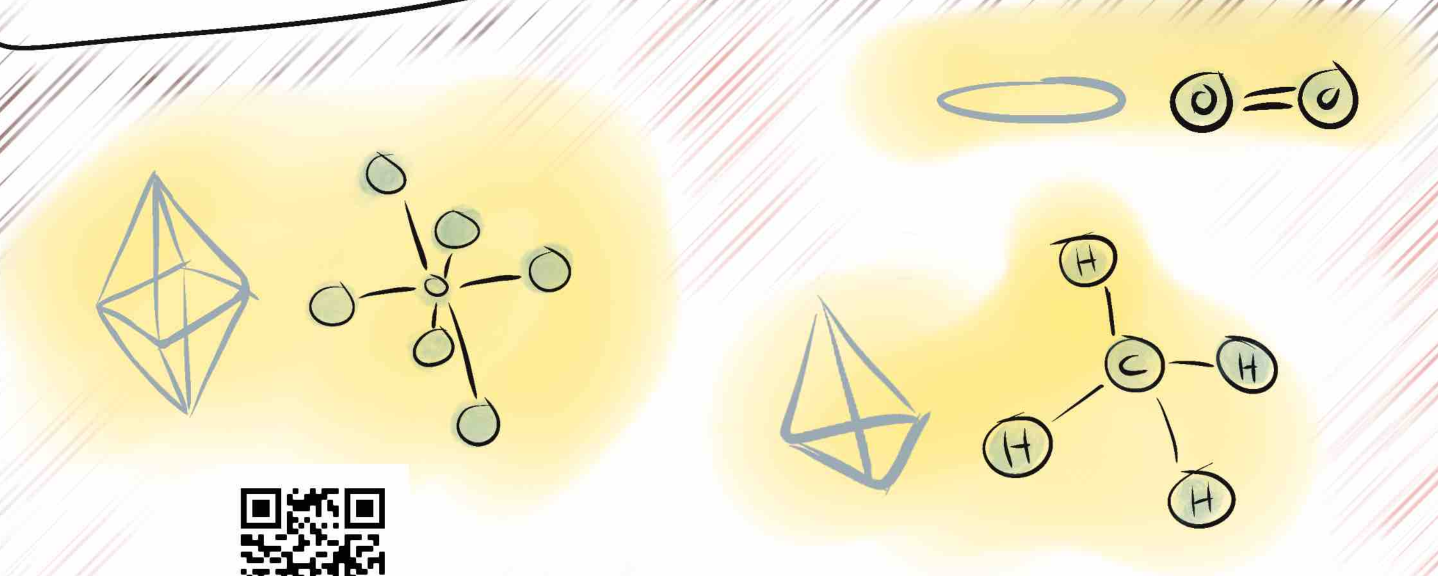

Bonding + shape

Copyright © Cook, 2022, CC by 4.0

Ionic Bonding

- electrostatic attraction between positive & negative ions
- usually METAL + non-metal
- atoms want full outer shell (8e-) by gaining or losing electrons

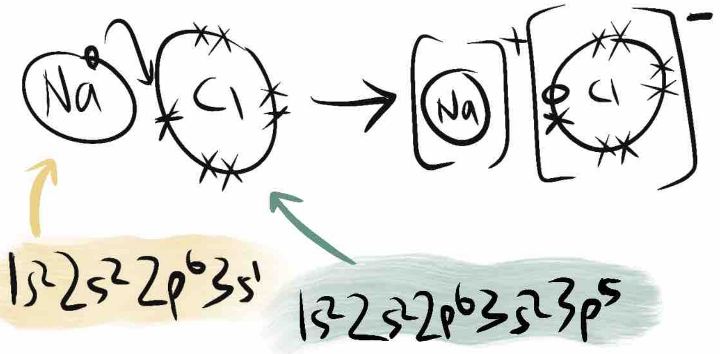

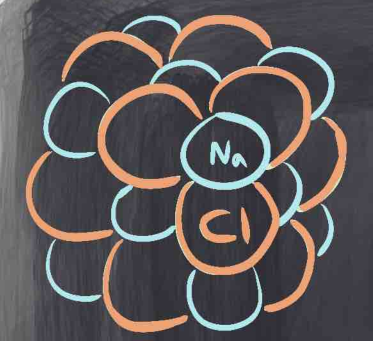

Form salt crystals of giant ionic lattices

Dot cross diagrams

* for outer shell electrons
* help show bonding

$Al \rightarrow Al^{3+} + 3e^-$

$3F + 3e^- \rightarrow 3F^-$

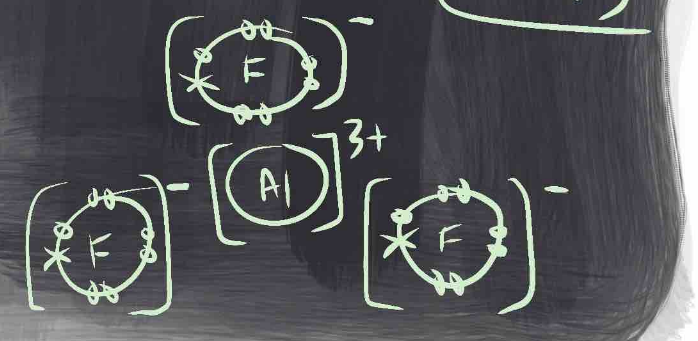

Covalent Bonding

- electrons shared between atoms to both gain full outer shell of electrons (overlap of orbitals)

H—H **1** shared pair = single bond

O=O **2** shared pairs = double bond

N≡N **3** shared pairs = triple bond

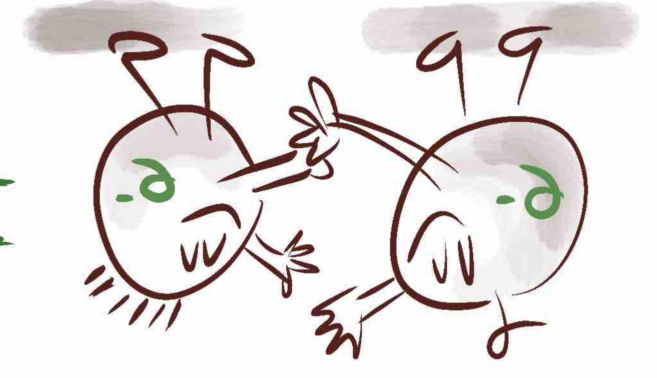

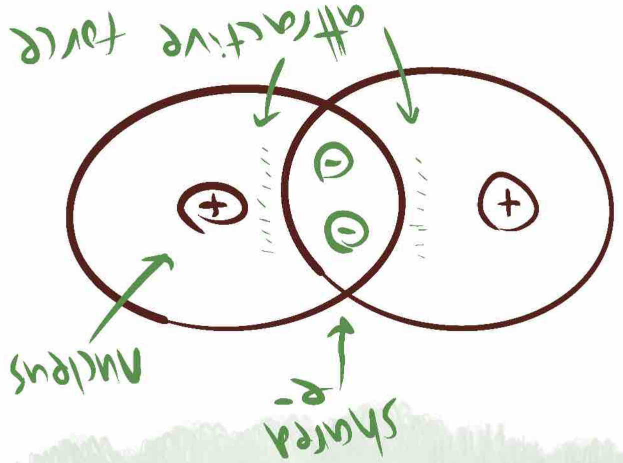

SHAPE

# Bonds	1	2	3	4	5	6
	linear	linear	trigonal planar	tetrahedral	trigonal bipyramidal	octahedral
eg	H_2	CO_2	BF_3	CH_4	PCl_5	SF_6

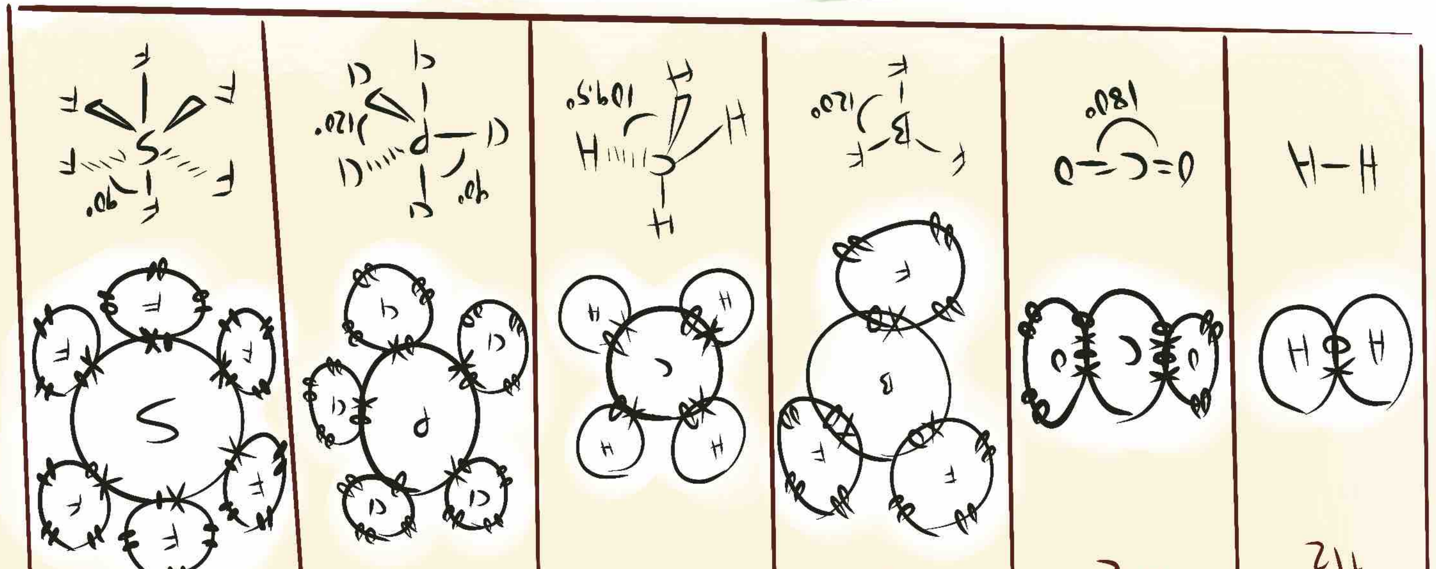

not normal (expansion of octet rule)

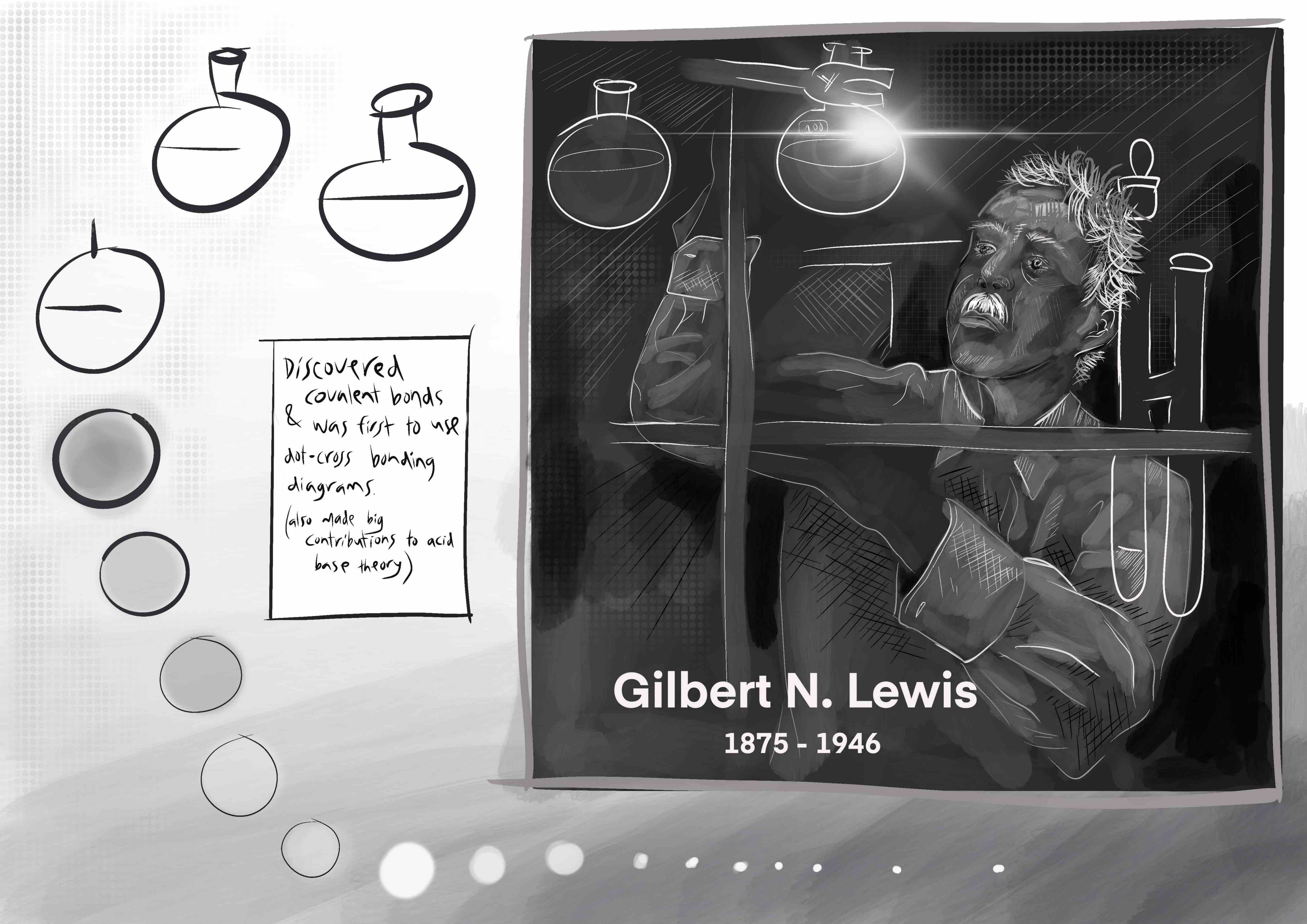
Discovered covalent bonds & was first to use dot-cross bonding diagrams.
(also made big contributions to acid base theory)
Gilbert N. Lewis
1875 - 1946

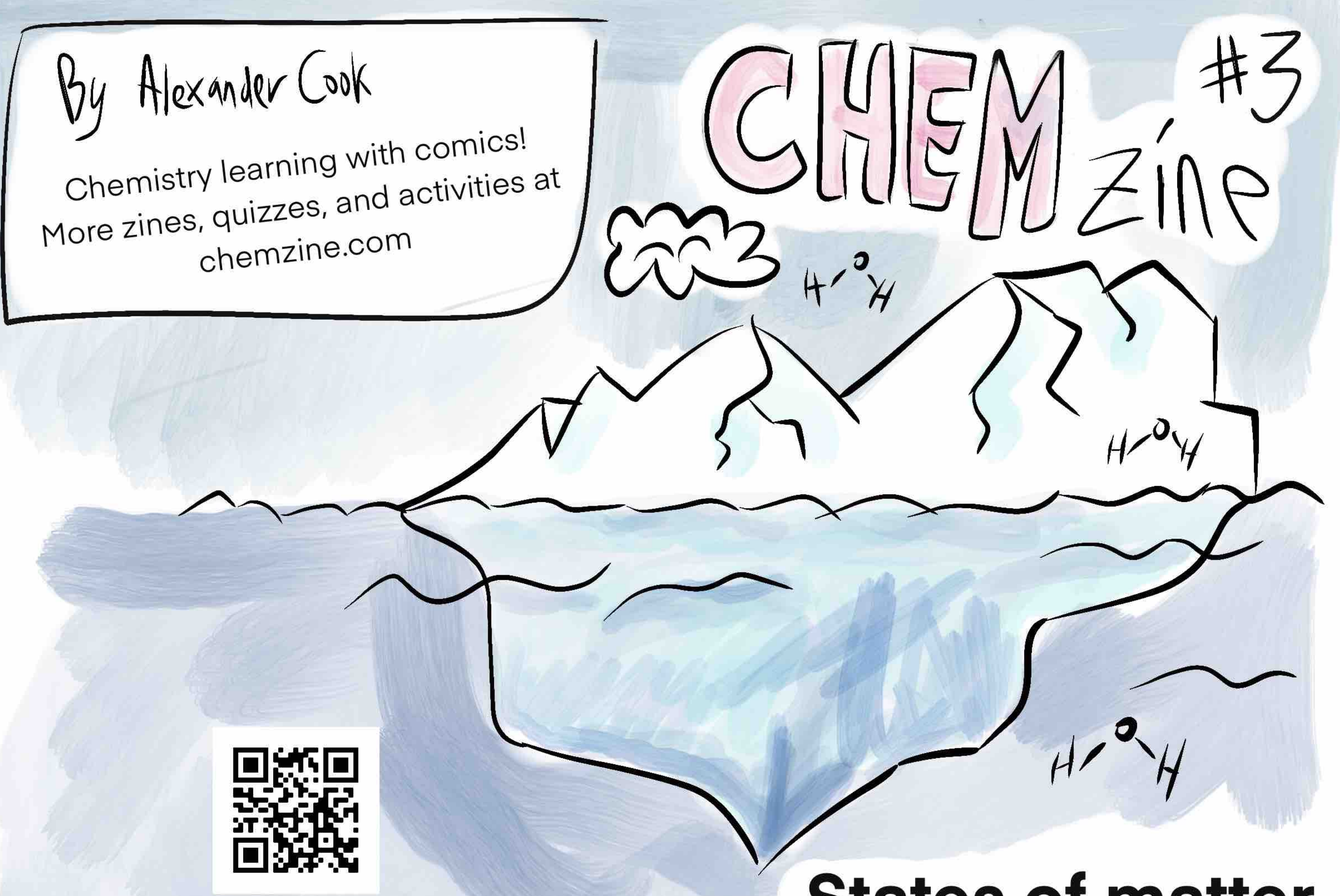

States of matter

By Alexander Cook

Chemistry learning with comics!
More zines, quizzes, and activities at
chemzine.com

Copyright © Cook, 2022, CC by 4.0

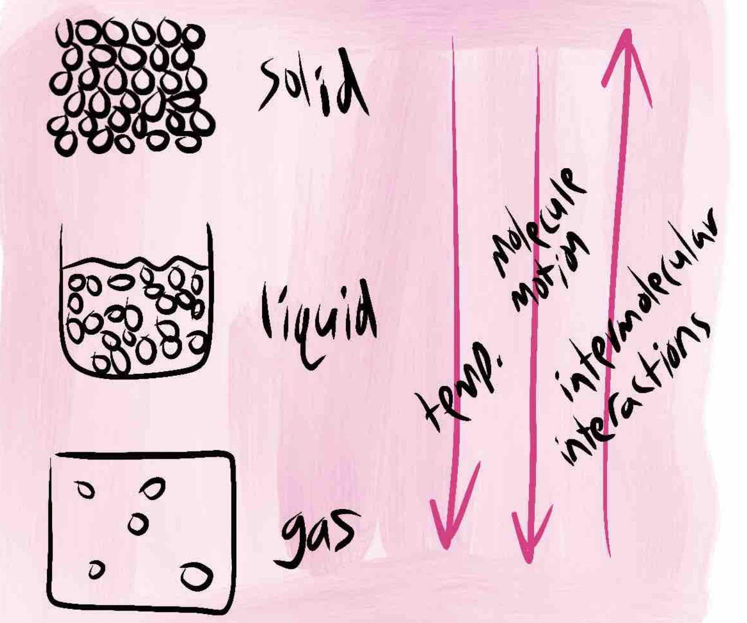

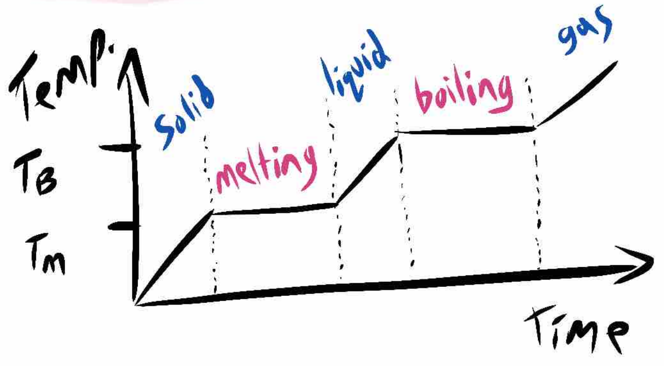

Intermolecular forces

elements in a molecule have different abilities to attract electrons (ie electrons not shared equally)

= ELECTRONEGATIVITY

⇩

Intermolecular forces

① Van der Waals

a) induced dipole – induced dipole
– weakest, non polar

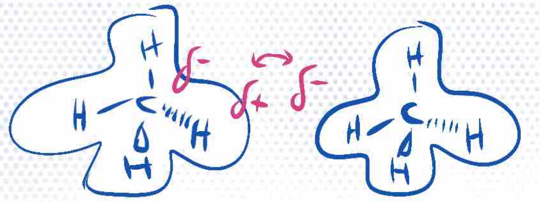

b) permanent dipole – permanent dipole
– polar molecules

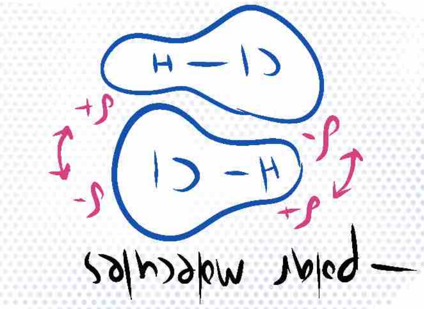

c) permanent dipole – induced dipole

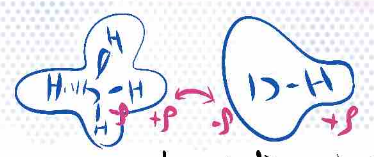

② Hydrogen bonding
– strongest, molecules with highly electronegative atoms (O, N, F, H)

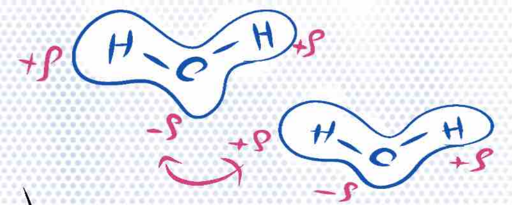

Gases

– very low density
– diffuse & fill shape of container

ideal gas → no intermolecular forces (not really true)
→ no energy loss in molecular collisions

$$pV = nRT$$

pressure (Pa)
volume (m^3)
moles mol ($n = \frac{m}{Mr}$)
gas const. (8.314 $J\,mol^{-1}K^{-1}$)
temp. (K)

Liquids

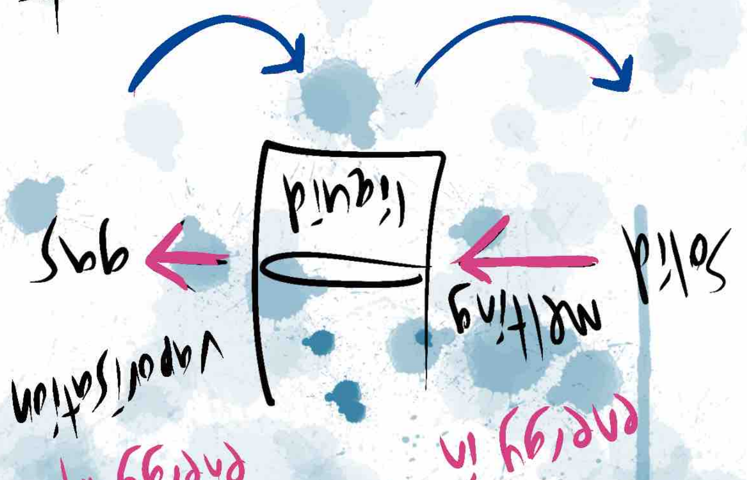

SOLIDS

ionic, covalent, metallic

giant covalent (covalent bonds in all directions → very strong materials)

simple covalent (only intermolecular forces → low melting point)

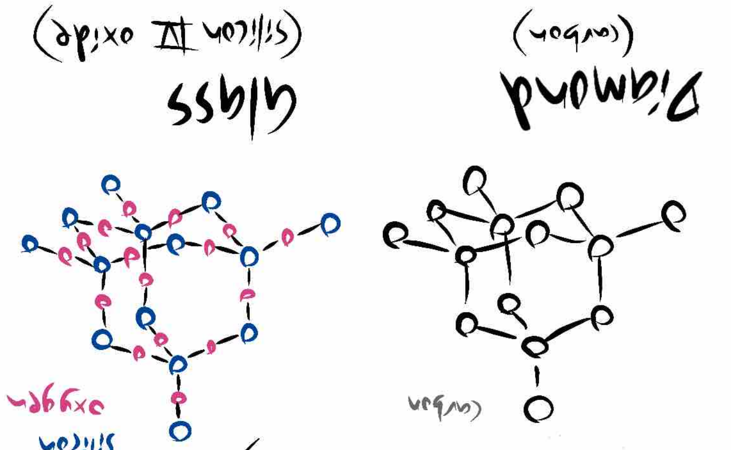

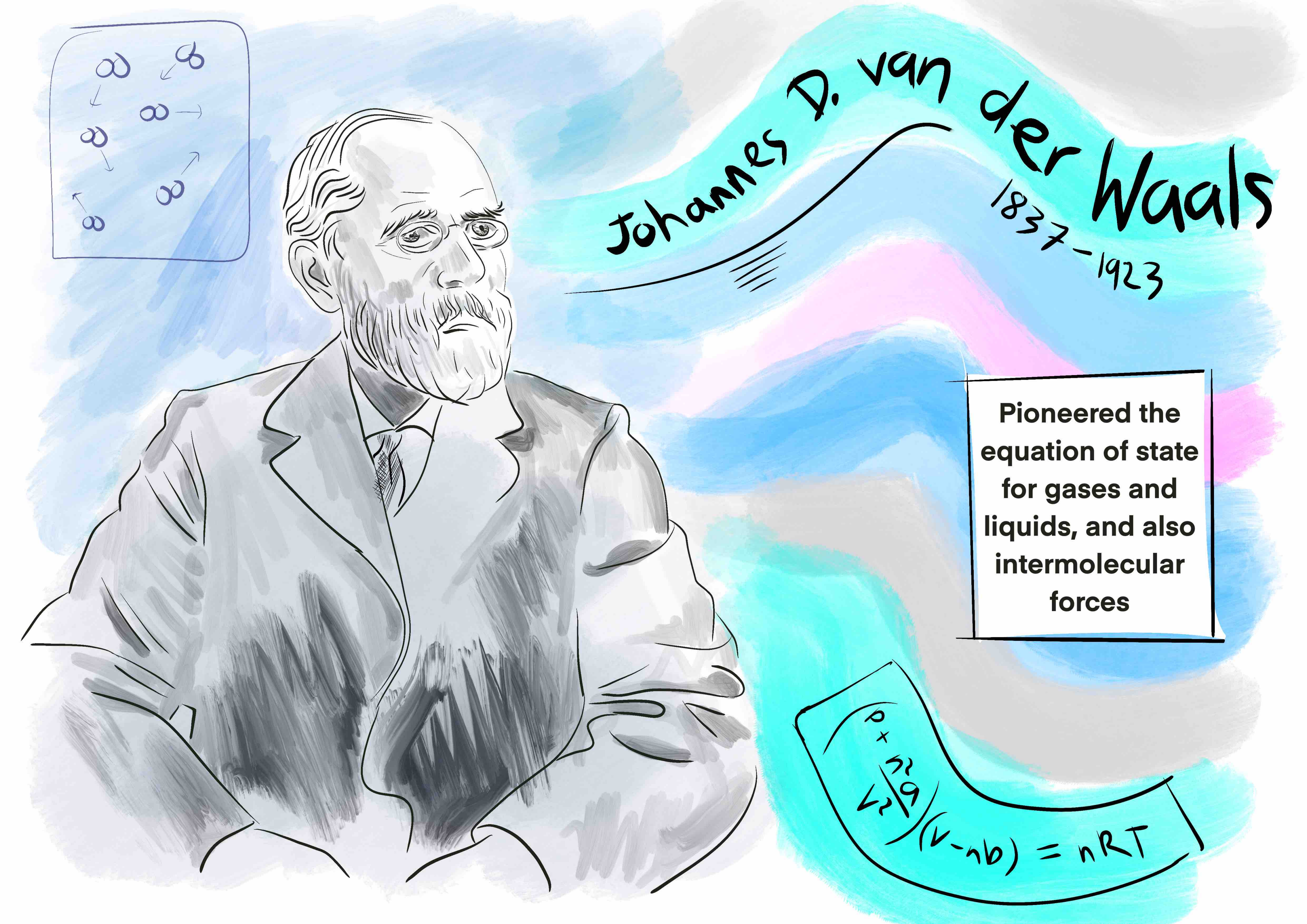
Johannes D. van der Waals
1837-1923
Pioneered the equation of state for gases and liquids, and also intermolecular forces
$\left(p + \frac{n^2 a}{V^2}\right)(V - nb) = nRT$

CHEM zine #4

By Alexander Cook

Chemistry learning with comics!
More zines, quizzes, and activities at chemzine.com

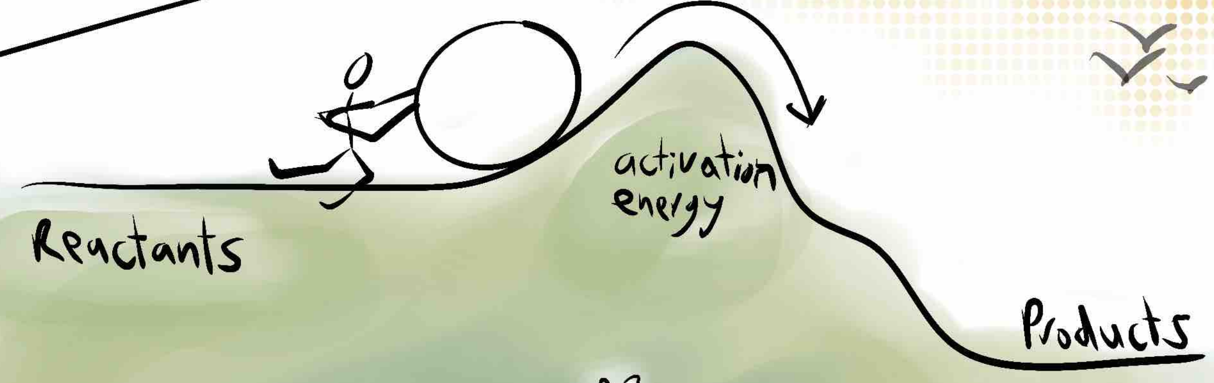

How fast? How far?

Copyright © Cook, 2022, CC by 4.0

Chemical reactions are accompanied by energy changes as bonds are broken & formed (usually in form of heat)

Exothermic gives off energy

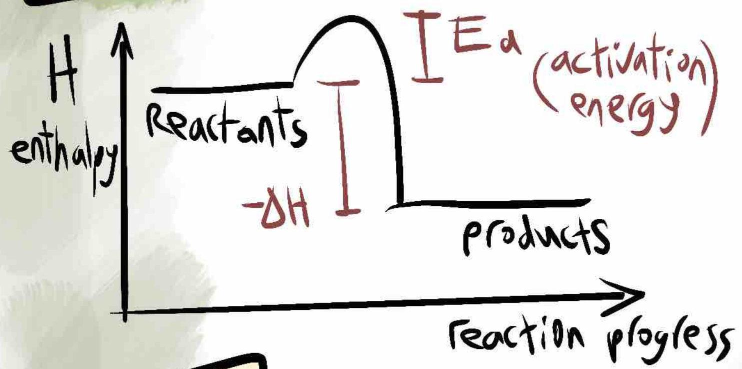

eg combustion

$CH_{4(g)} + 2O_{2(g)} \rightarrow CO_{2(g)} + 2H_2O_{(g)}$

$\Delta H^{\circ} = -852 \text{ kJ mol}^{-1}$

enthalpy change for one mole of substance completly burned in oxygen (at room temp and pressure)

Endothermic requires energy input

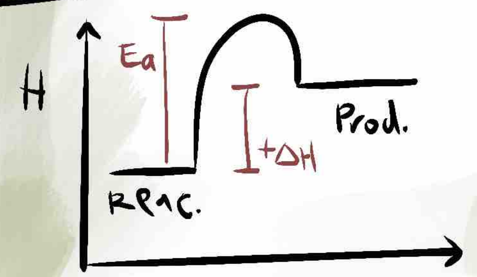

Hess's law

Enthalpy change independent of reaction route

$A+B \xrightarrow{\Delta_1 H} C$

$A+B \xrightarrow{\Delta_2 H} D \xrightarrow{\Delta_3 H} C$

$\Delta_1 H = \Delta_2 H + \Delta_3 H$

Reaction rate — How fast?

1) Temperature → Higher energy
2) Concentration → reactant collisions → more collisions
3) Catalyst → less energy required to reach products

Catalysts

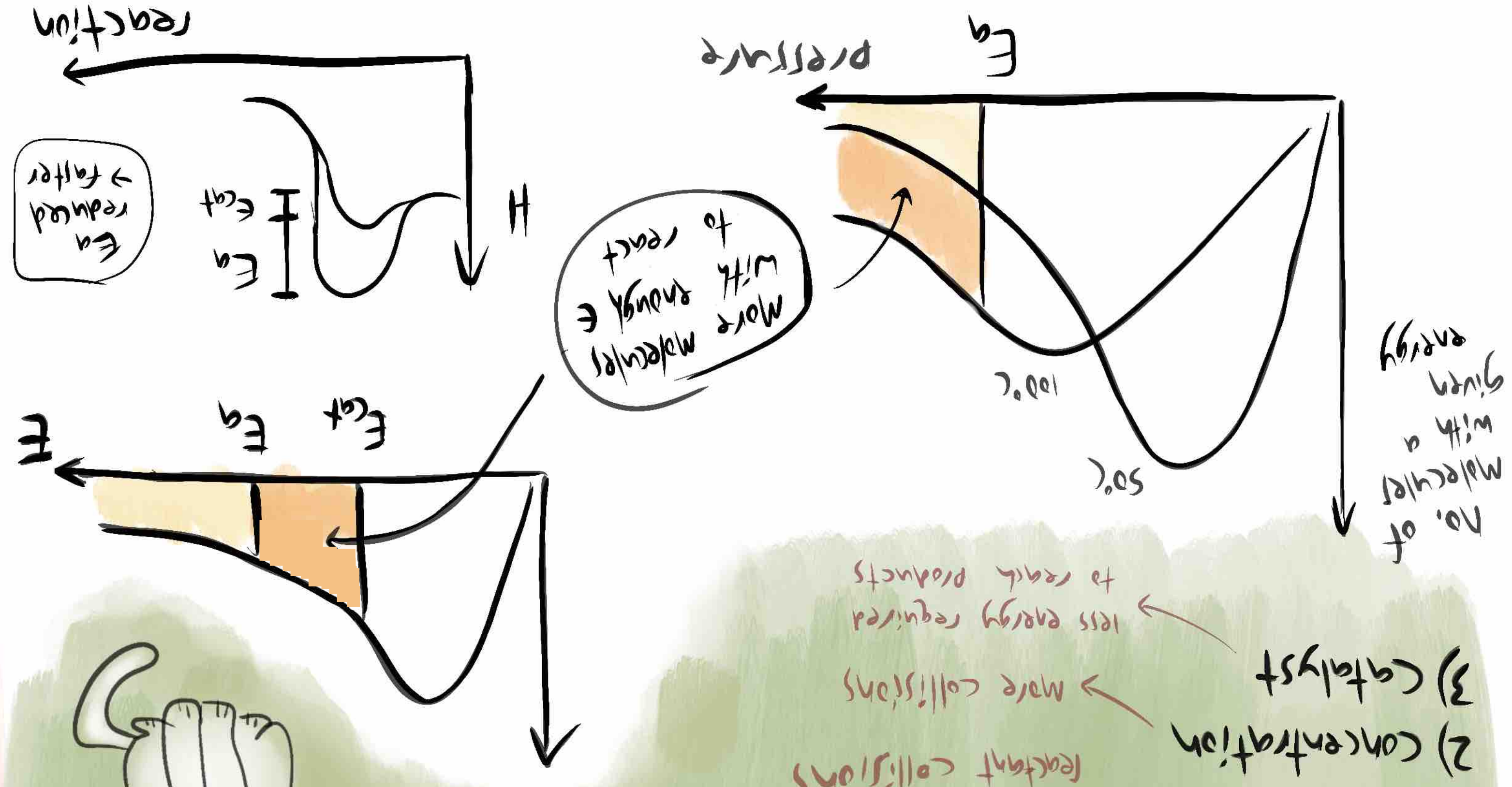

$2A + B \rightarrow C$

rate $= k[A]^2[B]^1$ (2nd order, 1st order; k = rate const.)

rate $= \frac{\text{change in conc.}}{\text{time}}$ mol dm^{-3} s^{-1}

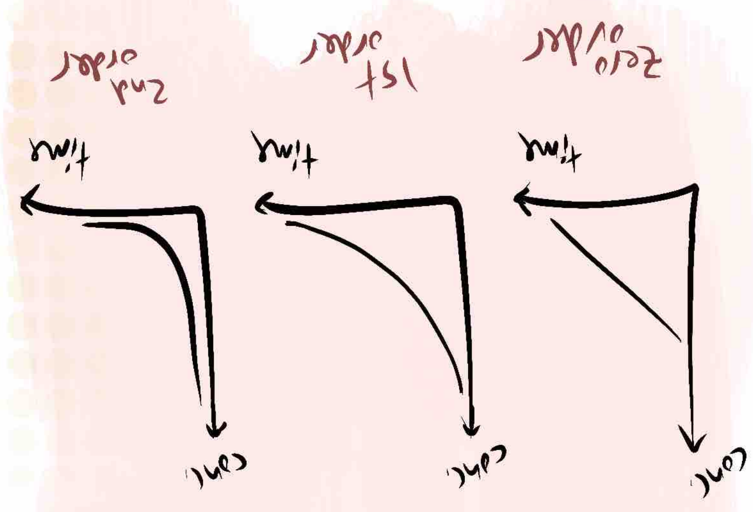

Equilibrium — How far?

forward & reverse rates in ballance

$N_{2(g)} + 3H_{2(g)} \rightleftharpoons 2NH_{3(g)}$

Equillibrium const. K_c ...equillibrium more towards reactants or products?

$K_c = \frac{\text{prod.}}{\text{react.}}$

$aA + bB \rightleftharpoons cC + dD$

$K_c = \frac{[C]^c[D]^d}{[A]^a[B]^b}$

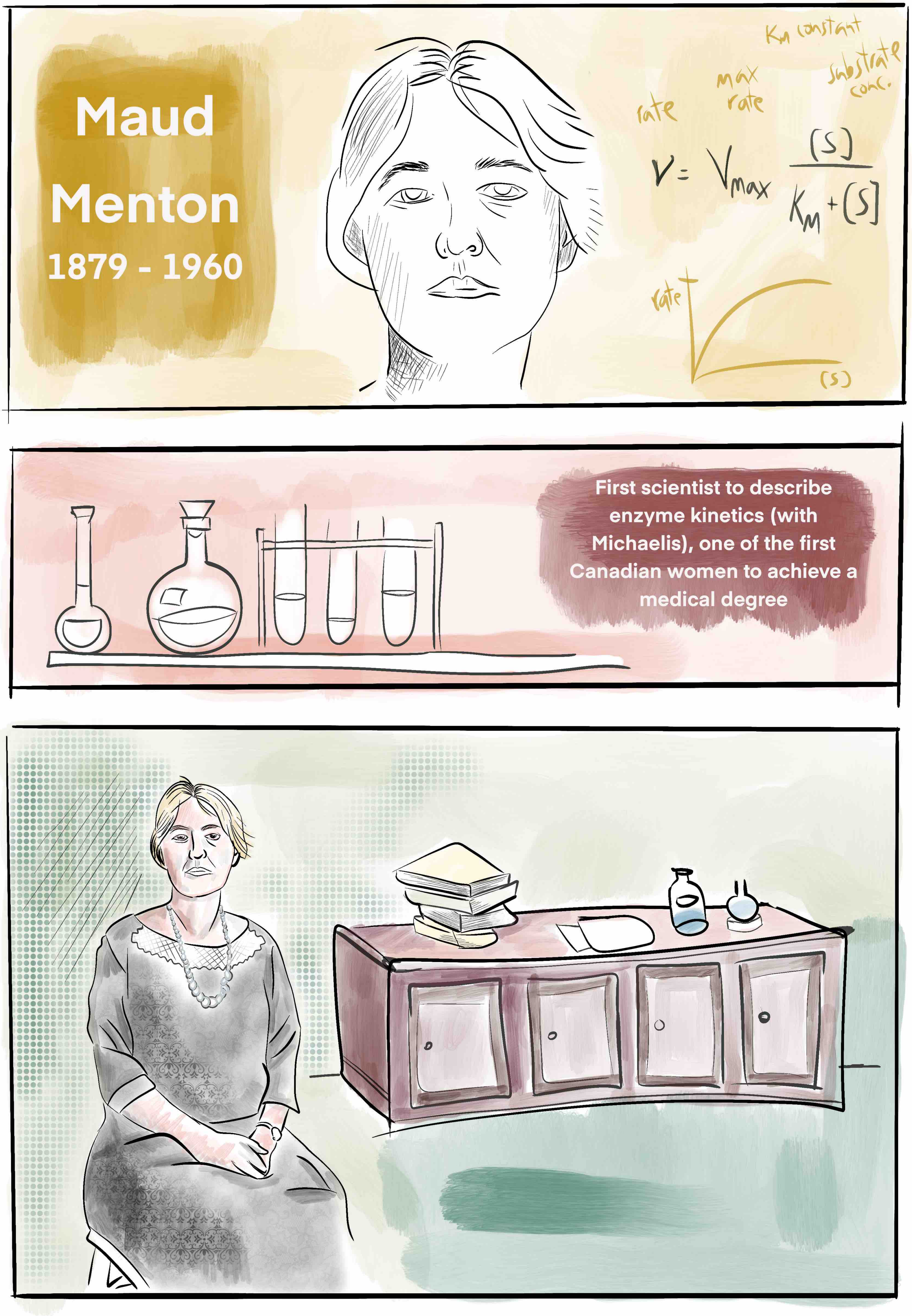
Maud
Menton
1879 - 1960
Km constant
substrate conc.
max rate
rate
v = Vmax (S) / Km + (S)
rate
(S)
First scientist to describe enzyme kinetics (with Michaelis), one of the first Canadian women to achieve a medical degree

Arenes

Substitution

Nitration

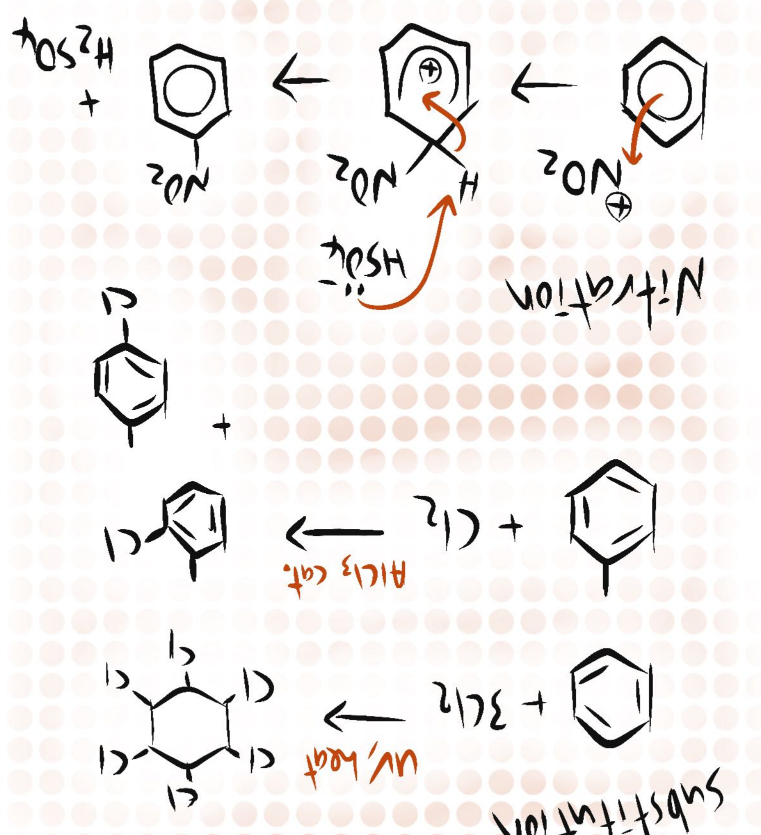

Carbocation stability

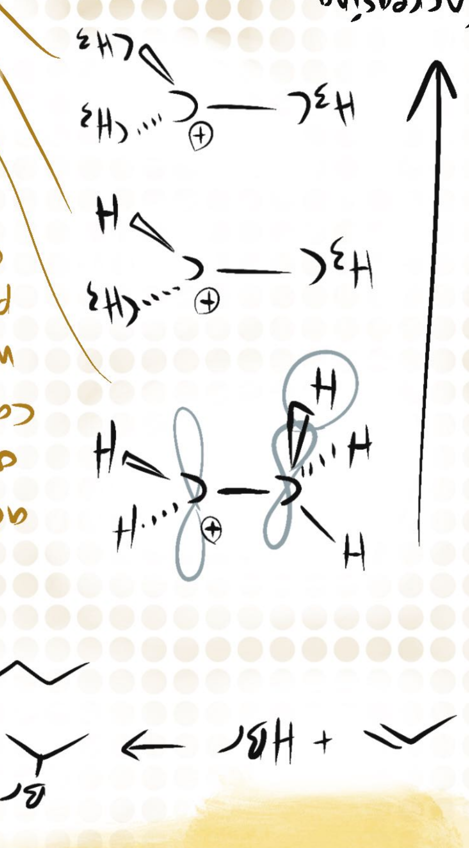

adjacent σ bond orbital can overlap with empty p orbital of carbocation = stability

1 overlap

2 overlaps

3 overlaps

Alkenes

all reactions below are electrophillic additions

$CH_2=CH_2 + H_2 \xrightarrow[\text{Ni cat.}]{150°C} CH_3CH_3$

$CH_3\text{-}CH=CH_2 + H_2O \xrightarrow[300°C,\ 65\,ATM]{HPO_3} CH_2CH_2CH_2OH$ or $CH_3CH(OH)CH_3$

$CH_3\text{-}CH=CH_2 + H\text{-}Br \longrightarrow CH_3CHBrCH_3$

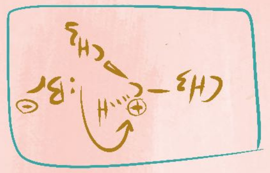

mechanism (arrows show path of electrons)

$CH_2=CH_2 + Br_2 \longrightarrow CH_2BrCH_2Br$

same mechanism as above

aqueous bromine colour change 'test' for alkenes (orange to colourless)

Isomerism

same molecule but different arrangment of atoms

i) chain isomerism

ii) Position isomerism

iii) Stereoisomerism

Diastereomers

enantiomers

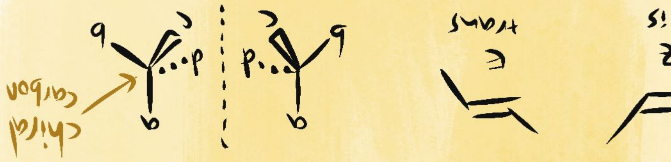

due to restricted rotation around double bond.

4 different groups on one carbon ∴ non-superimposable mirror images

By Alexander Cook

Chemistry learning with comics!
More zines, quizzes, and activities at chemzine.com

CHEMzine

#5

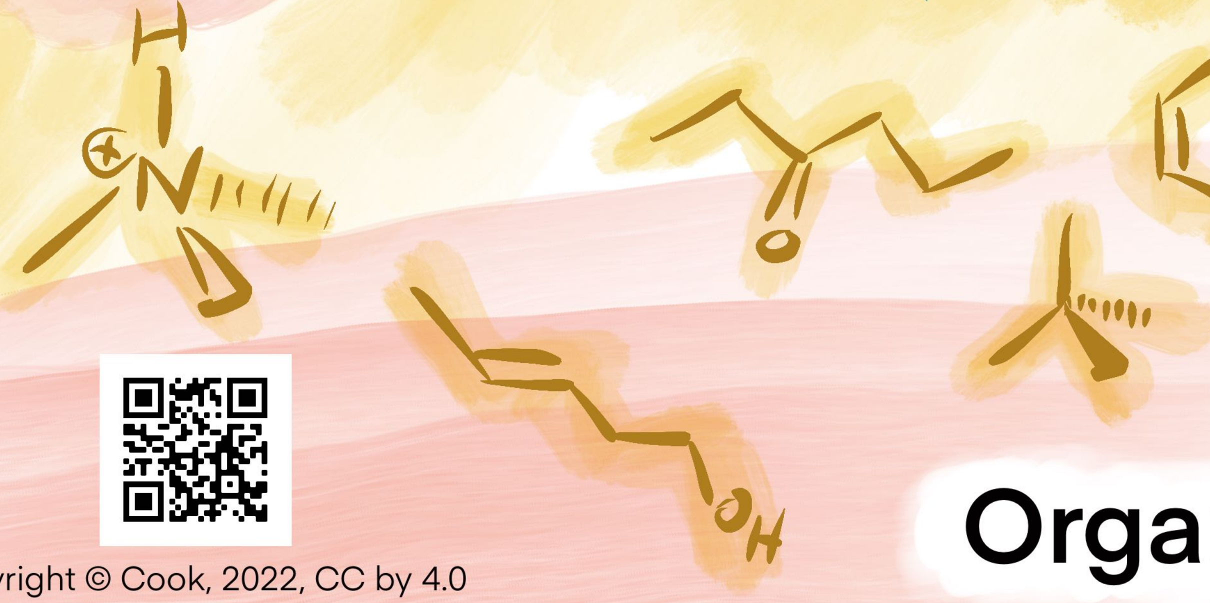

Organic

Copyright © Cook, 2022, CC by 4.0

Formula

Structural: $CH_3CH_2CH(OH)CH_3$

Displayed

Skeletal

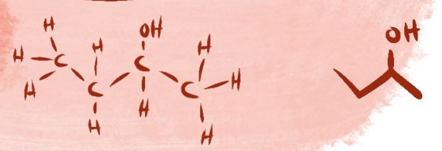

Nomenclature

# carbons	stem begins
1	meth-
2	eth-
3	prop-
4	but-
5	pent-
6	hex-

bond	stem end
C-C	an
C=C	en
2× C=C	dien
3× C=C	trien
C≡C	yn

suffix	group
-al	aldehyde
-one	ketone
-ol	-OH alcohol
-oic acid	carboxylic acid

eg $CH_3CH_2CH(OH)CH_3$ Butan-2-ol, CH_2ClCH_2CHO 3-chloropropanal

Types of reaction

nucleophillic (Towards +ve nucleus)

electrophillic (Towards electron rich centre)

- addition
- substitution
- elimination
- hydrolysis
- oxidation
- reduction

Bond hybridisation

e^- in orbital	bond formed
s + s	σ
p + p	π
s + p	σ

but hybridised (with mixture of s & p characteristics and different angles)

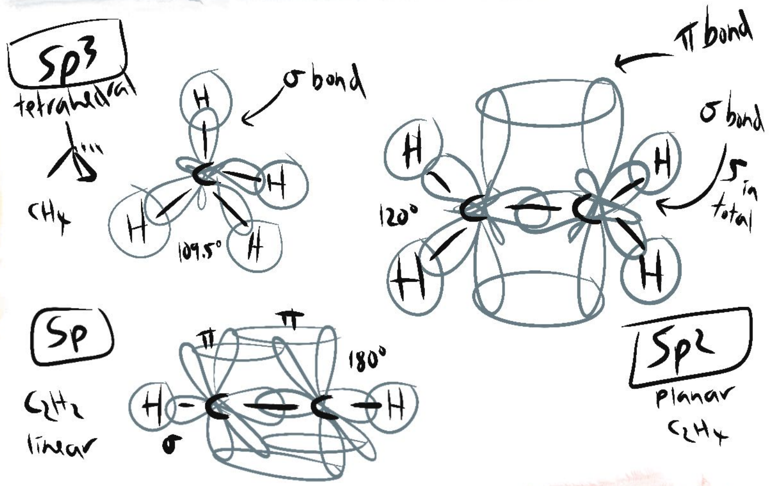

Benzene

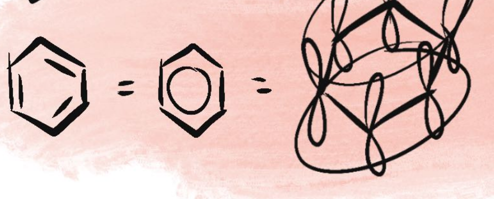

cloud of electrons from overlapping p orbitals (above & below)

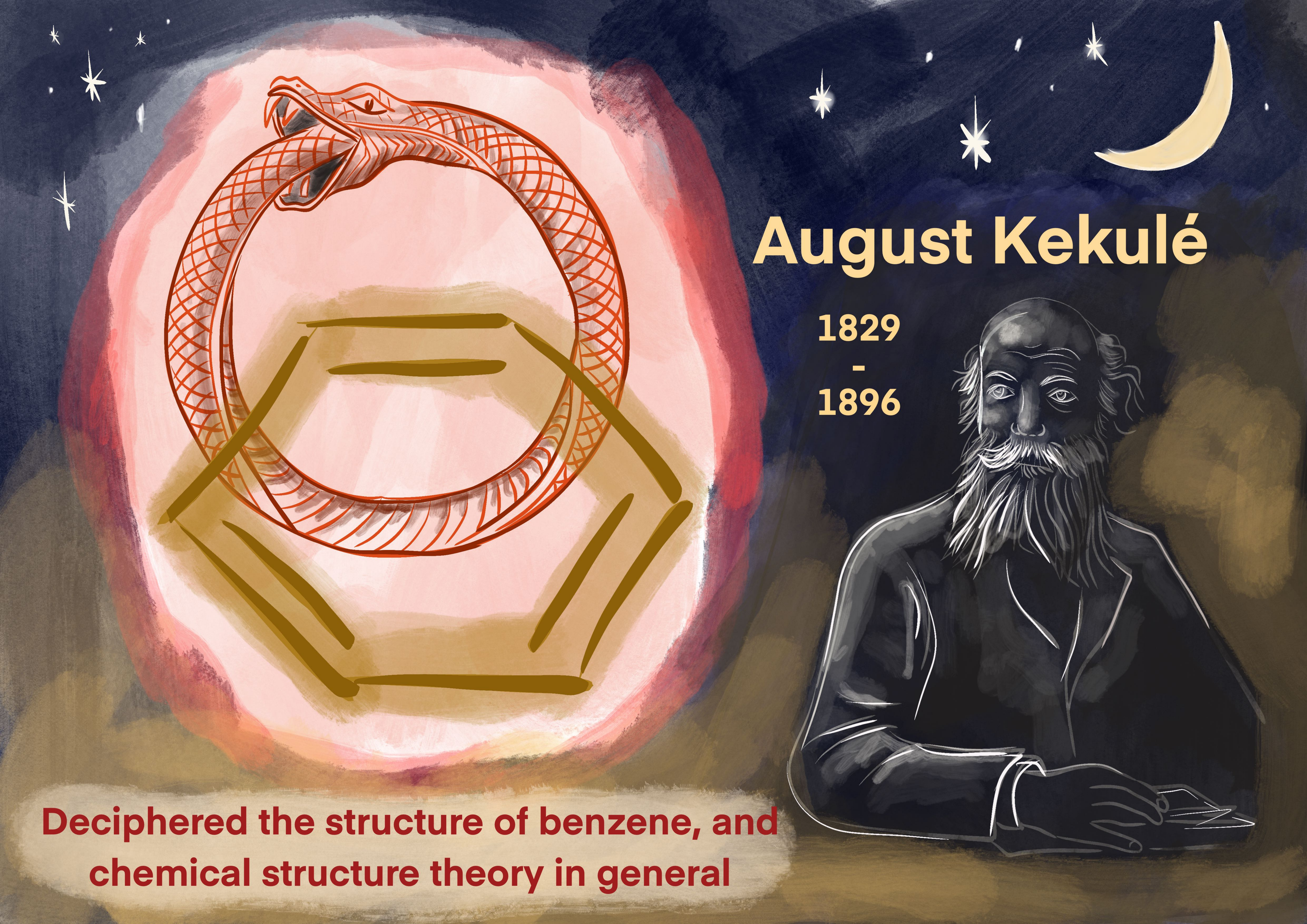

August Kekulé
1829
-
1896
Deciphered the structure of benzene, and chemical structure theory in general

CHEM zine #6

More organic

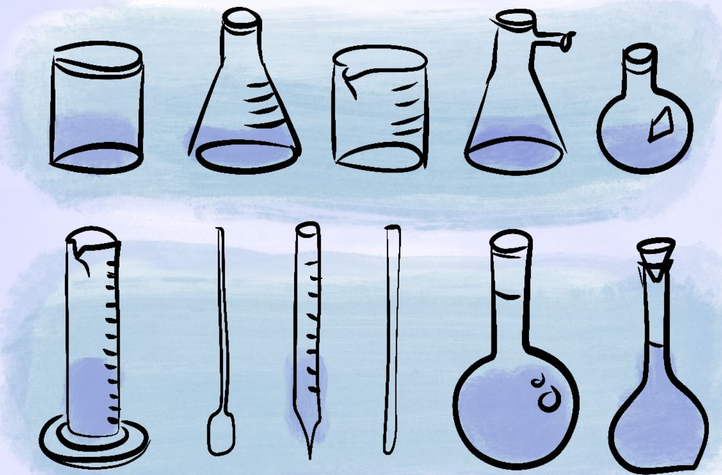

By Alexander Cook

Chemistry learning with comics!
More zines, quizzes, and activities at
chemzine.com

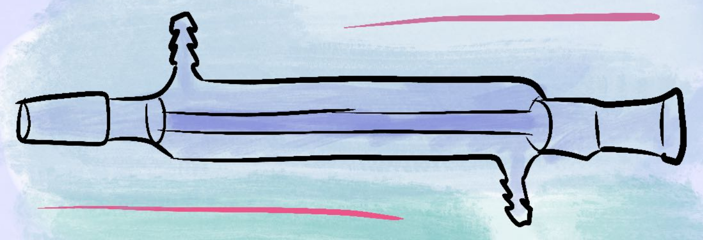

Copyright © Cook, 2022, CC by 4.0

Halogenoalkanes

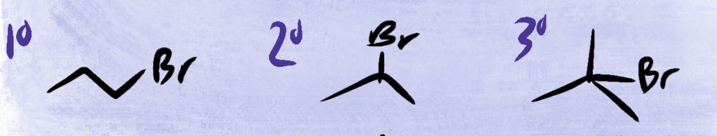

undergo nucleophilic substitution reactions due to polar C-Br bond

Primary → SN2

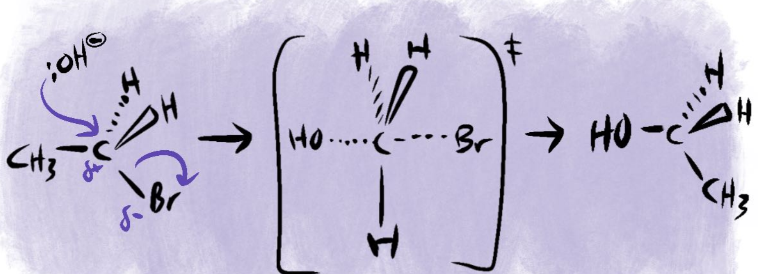

transition state

Secondary → SN2/SN1 mixture

tertiary → SN1

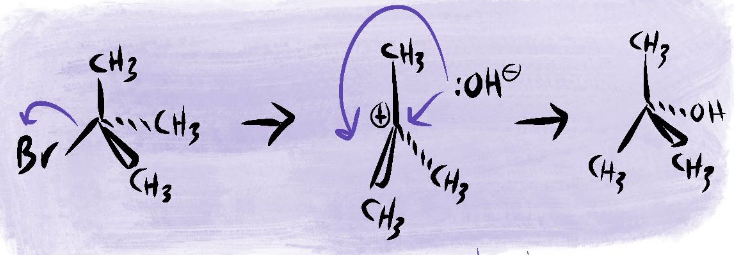

OH can attack from either side

Elimination

HCl + OH⁻ → H₂O + Cl⁻

CH₃CH₂Br + OH⁻ → H₂O + CH₂=CH₂ + Br⁻

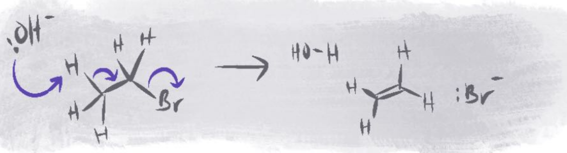

Alcohols

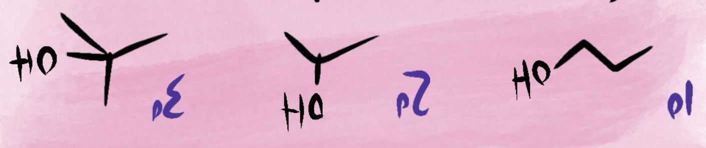

primary secondary tertiary

Formation

- Electrophillic addition of steam to alkene (H_3PO_4 catalyst).
- Substitution of haloalkane (NaOH(aq), ΔT).
- Reduction of ketones ($NaBH_4$).
- Reduction of carboxylic acids ($LiAlH_4$).
- Hydrolysis of esters.

Reactions

- Combustion
  $CH_3CH_2OH + 3O_2 \rightarrow 2CO_2 + 3H_2O$
- Substitution
  $CH_3CH_2OH + HBr \rightarrow CH_3CH_2Br + H_2O$
  $CH_3CH_2OH + PCl_5 \rightarrow CH_3CH_2Cl + POCl_3 + HCl$
- Oxidation ($K_2Cr_2O_7$)

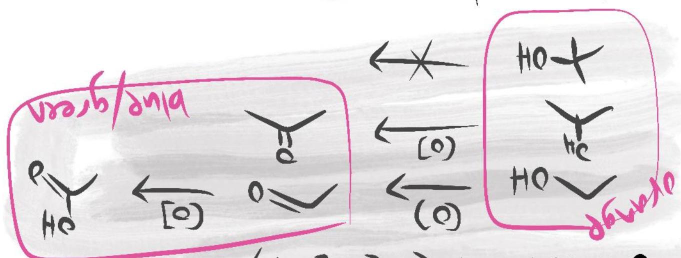

- Dehydration

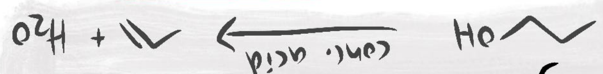

Carbonyl compounds

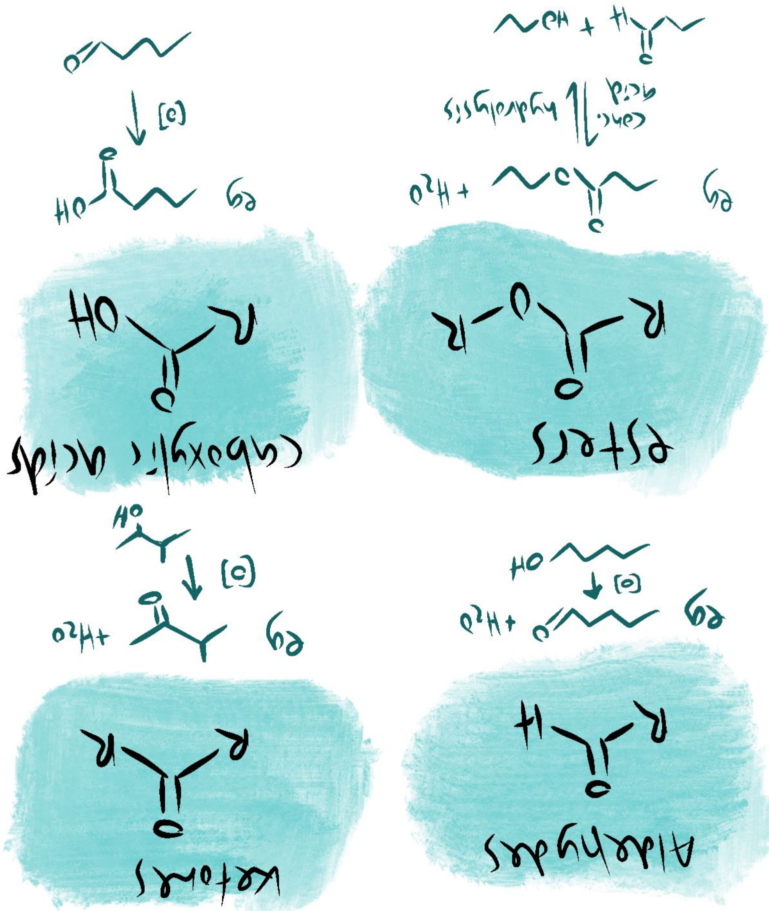

Nitrogen compounds

formation

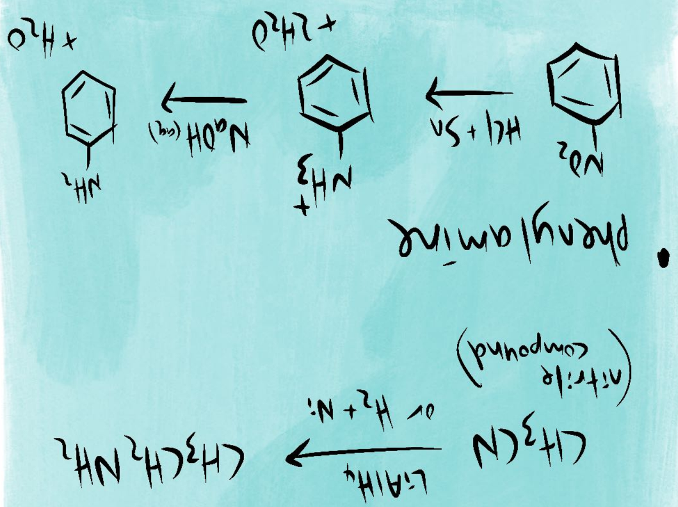

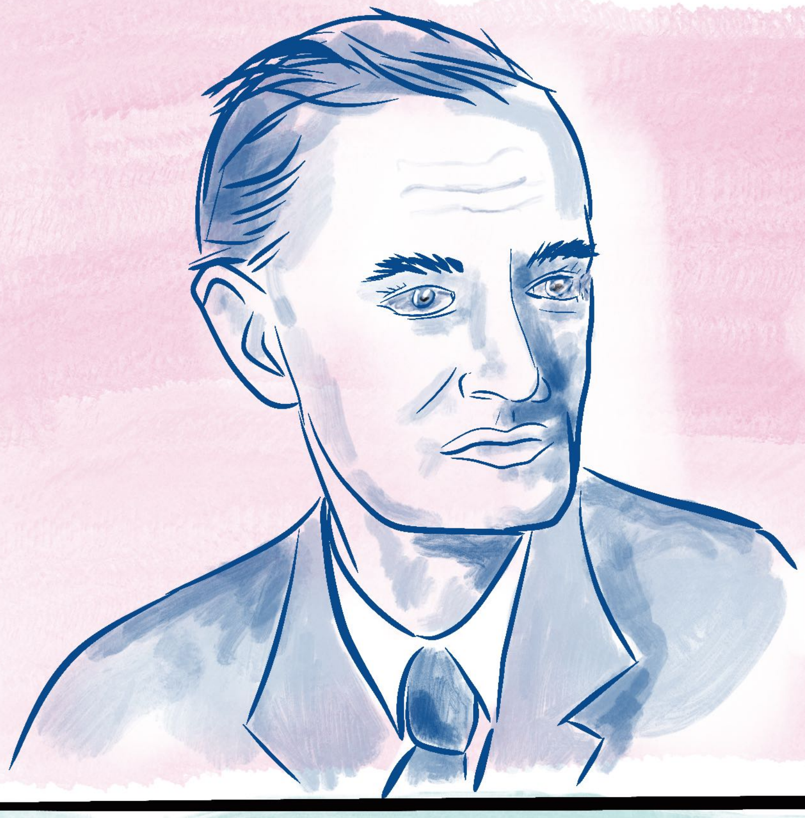

Christopher Ingold

1893 - 1970

Pioneered our understanding of the electronic basis of structure and reaction mechanism in organic chemistry - fundamental to modern science

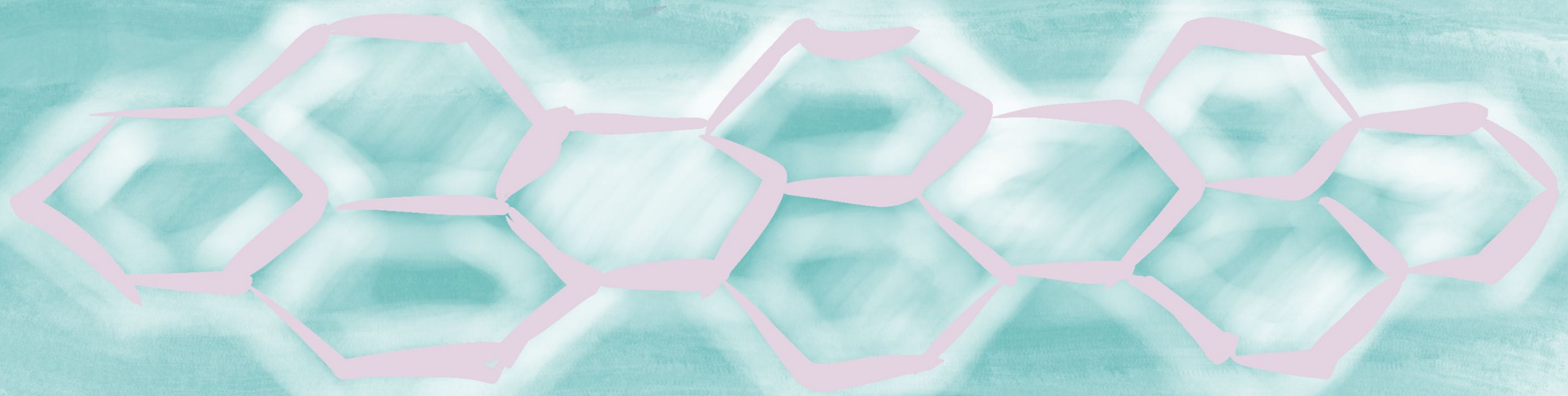

SN1

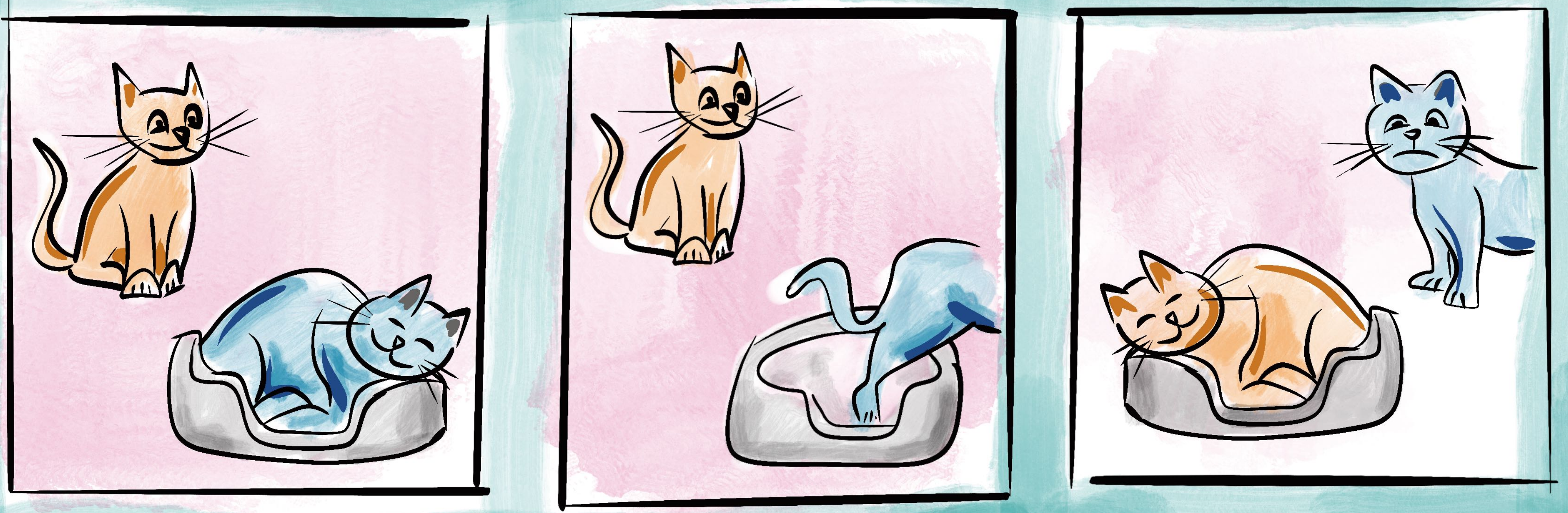

SN2

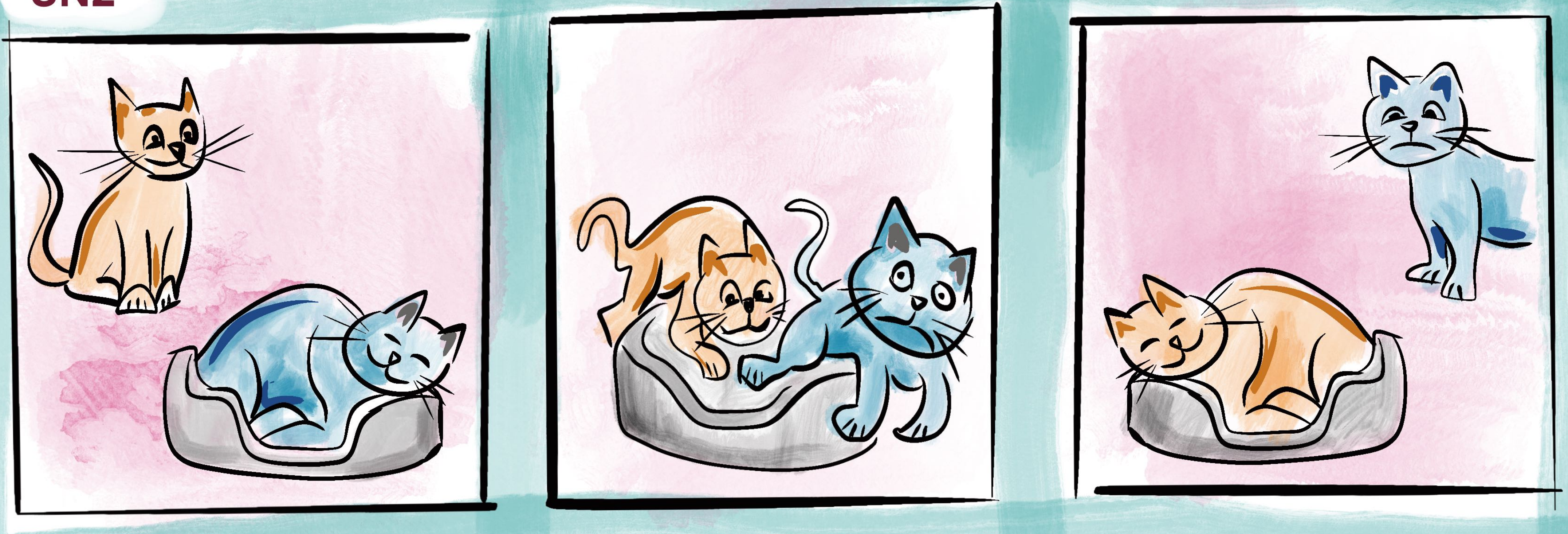

Polymer chemistry

By Alexander Cook

Chemistry learning with comics!
More zines, quizzes, and activities at chemzine.com

CHEMzine #7

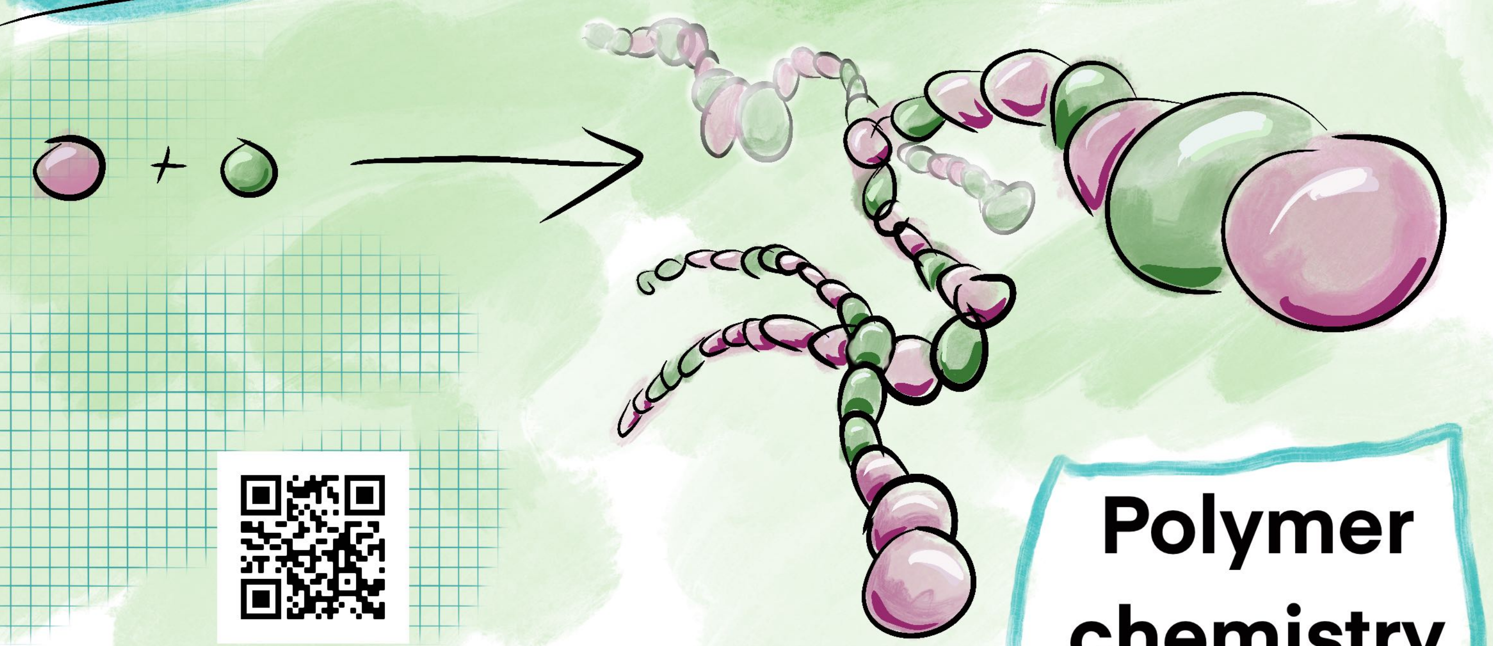

Copyright © Cook, 2022, CC by 4.0

Polymers

- large molecules with repeating subunits
- can be both natural & synthetic
- chain entanglements = unique material properties

Polymerisation

Kinetics: step growth | chain growth

Propagation mechanism:
- step growth: addition polymerisation, condensation polymerisation
- chain growth: radical, coordinative, cationic/anionic, ring opening

step growth

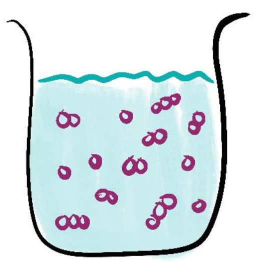

direct combination of monomers (only reach high molecular weight at high reaction conversion)

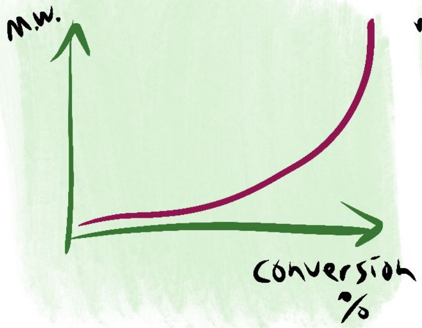

chain growth

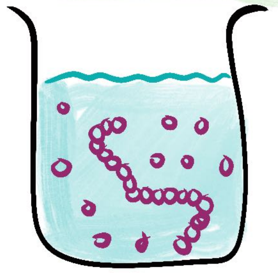

addition of monomers one at a time to chain end.

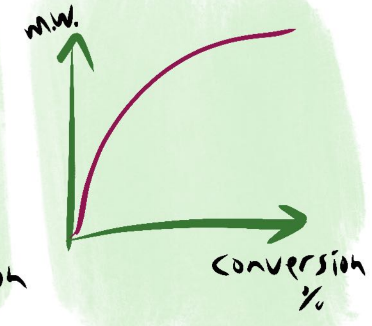

Addition polymers

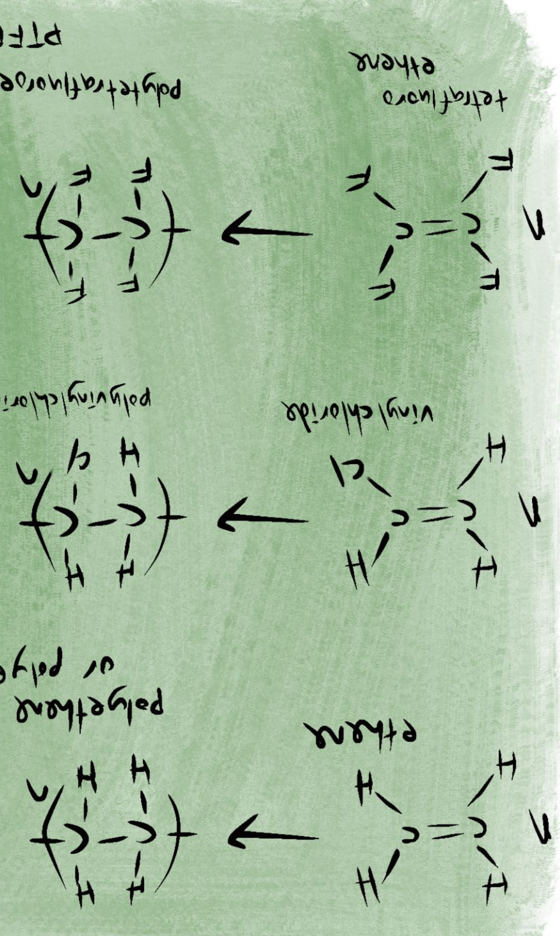

Condensation polymers

usually polyesters or polyamides, made with loss of a molecule of H_2O or HCl

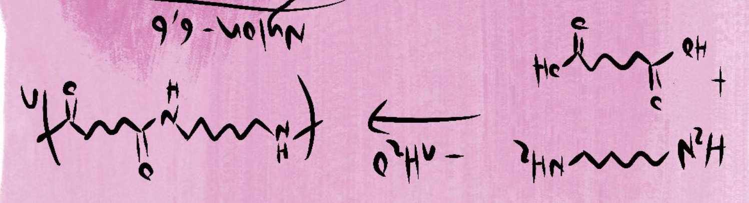

polyamides less degradable than polyesters which can hydrolyse back to monomers

Kevlar (bulletproof vests)

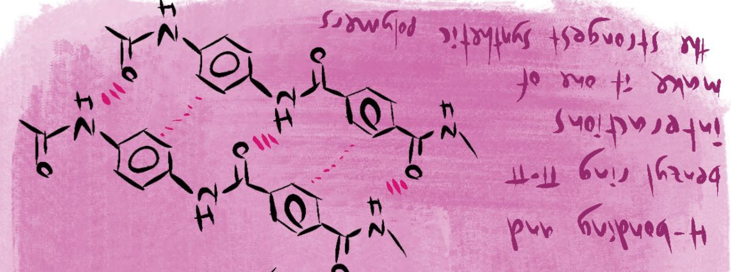

H-bonding and benzyl ring π-π interactions make it one of the strongest synthetic polymers

Radical polymerisations

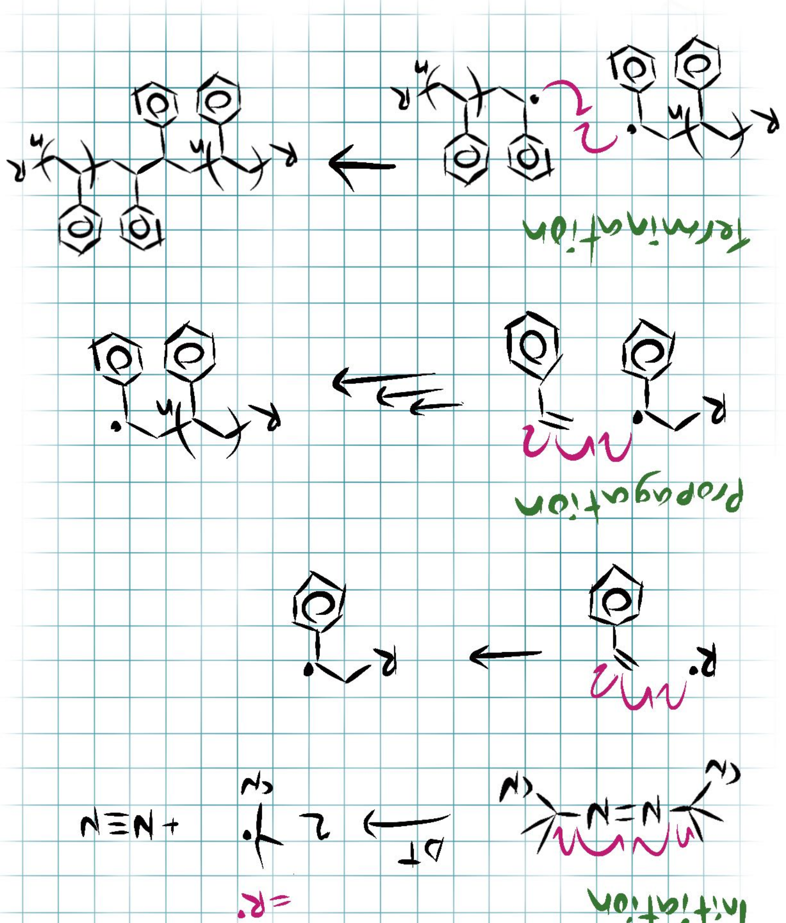

Physical properties

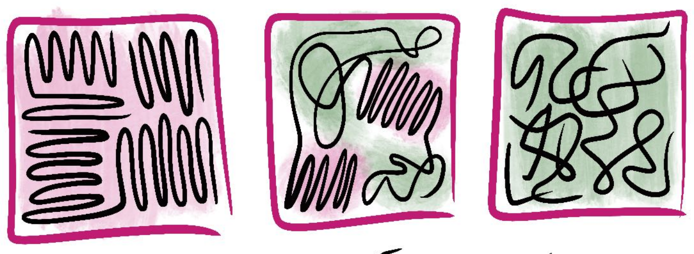

- low melting point
- easily deformed

- high melting point
- Rigid
- high tensile strength

Crosslinking

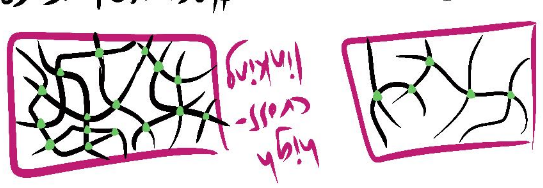

low cross-linking:
- elastomer
- easily deformed
- stretchy

high cross-linking:
- thermoset or resin
- hard
- brittle

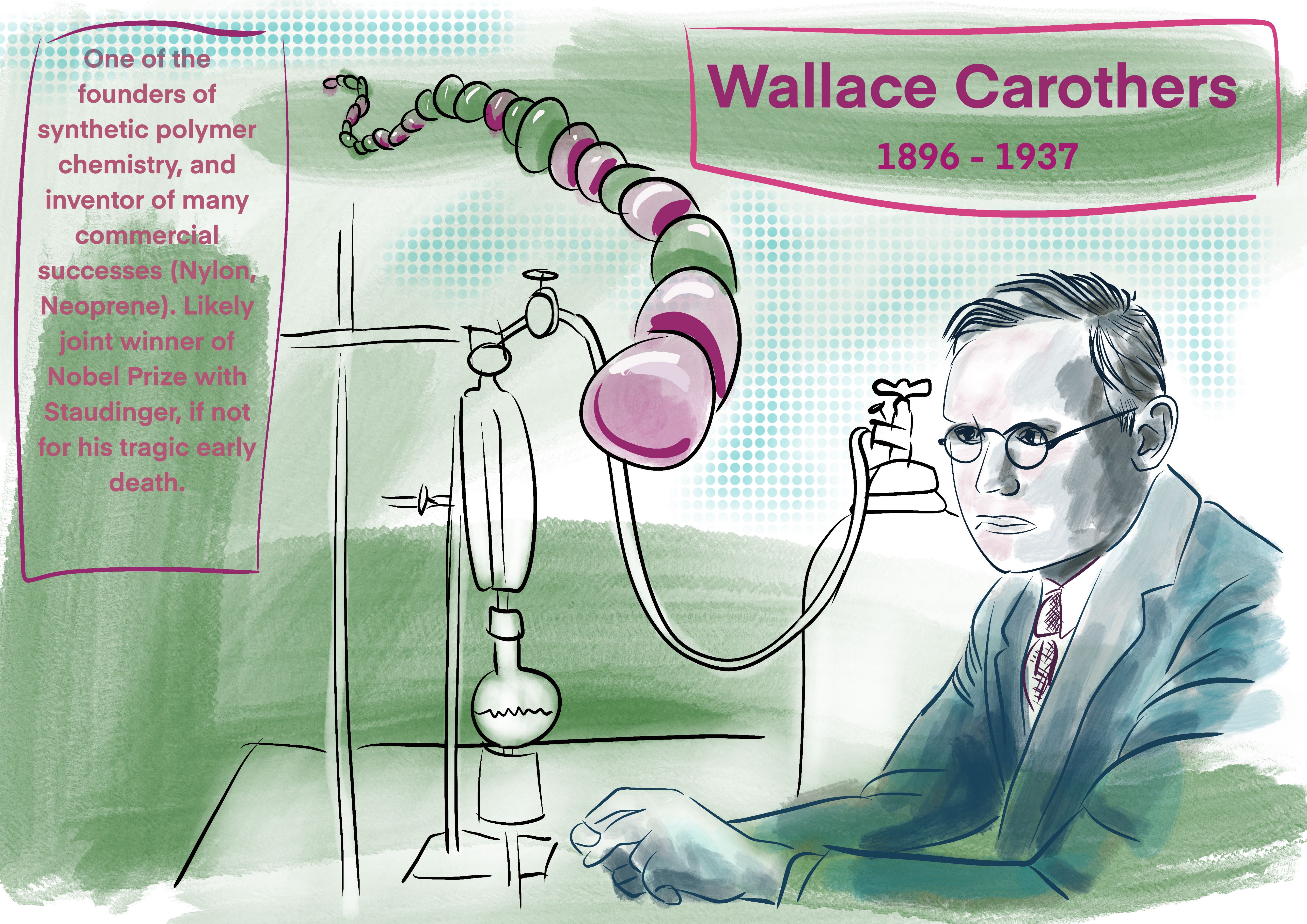

Wallace Carothers
1896 - 1937
One of the founders of synthetic polymer chemistry, and inventor of many commercial successes (Nylon, Neoprene). Likely joint winner of Nobel Prize with Staudinger, if not for his tragic early death.

By Alexander Cook

Chemistry learning with comics!
More zines, quizzes, and activities at
chemzine.com

CHEMzine #8

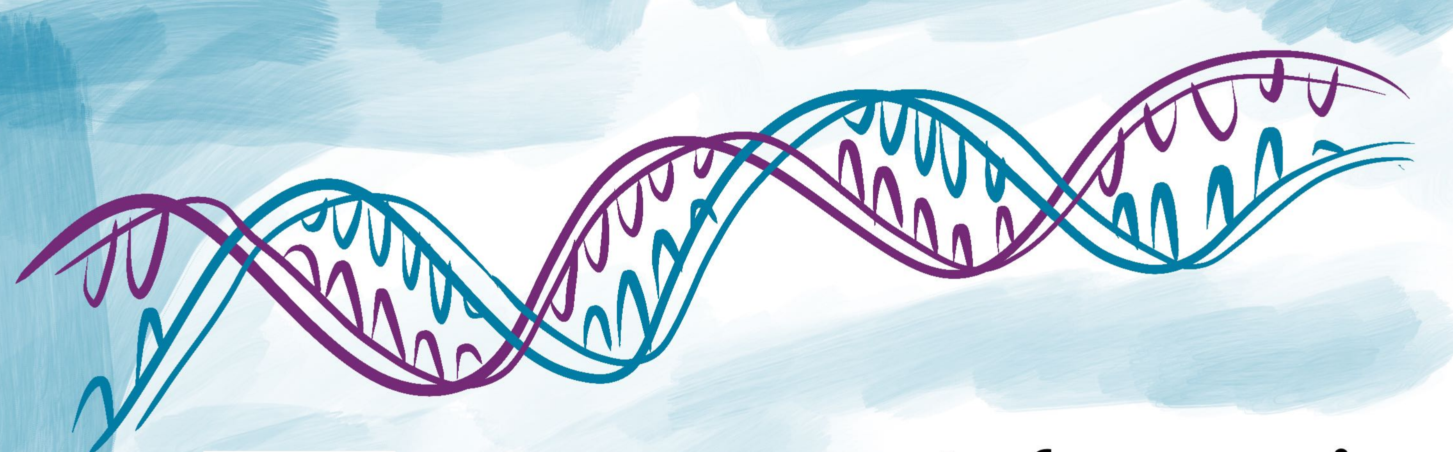

Information of life

Copyright © Cook, 2022, CC by 4.0

DNA deoxyribosnucleic acid

is a biological polymer made up of monomers called nucleotides. The monomers in turn have three components:

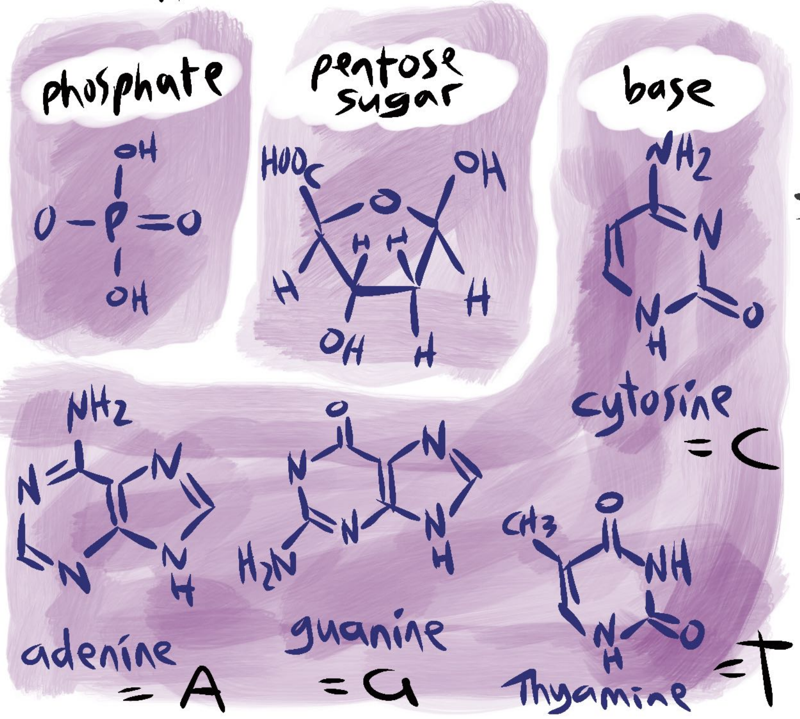

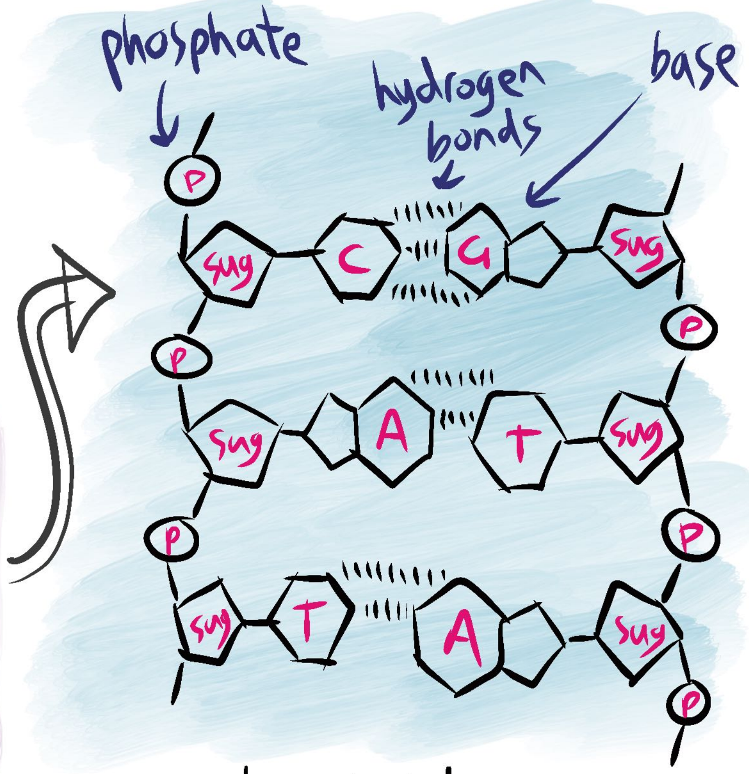

Two polynucleotide chains can form a double helix together due to hydrogen bonds between base pairs. (always CG & AT)

DNA base pair code controls the passing of genetic information in species & codes for protein synthesis in our cells.

RNA ribonucleic acid

DNA	RNA
Deoxyribose	Ribose sugar
ACGT bases	ACGU bases
Double strand	Single strand

RNA sequence codes for proteins

eg GAA = Glutamate Amino Acid, CAC = Histidine

CELL

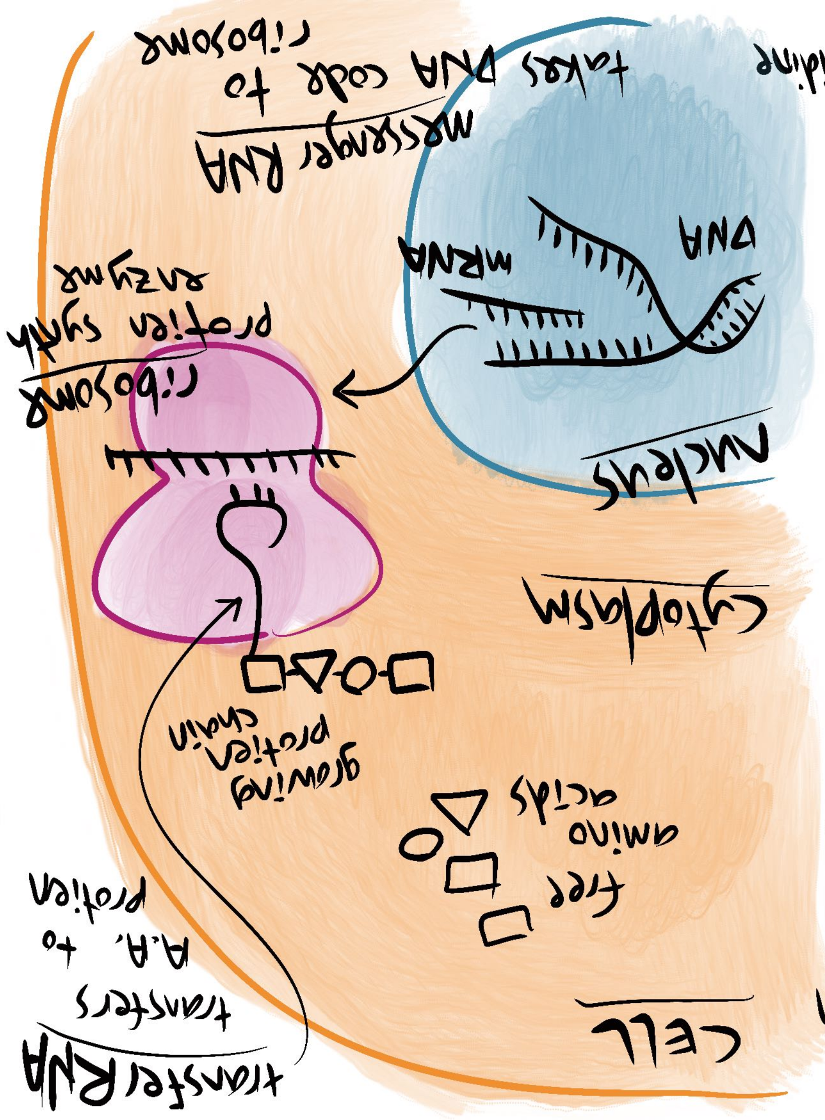

Cancer

Over time mutations in our DNA code can cause cells to divide & reproduce uncontrollably (and cause tumours)

anticancer drugs or chemotherapies stop these cancer cells from reproducing

eg cisplatin

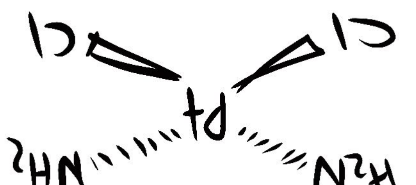

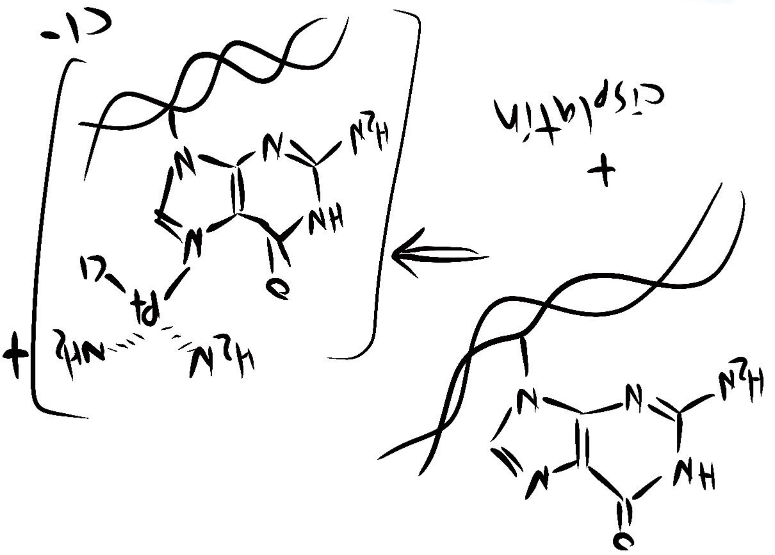

N from guanine substitutes a Cl on cisplatin (up to two times)

the disruption in the DNA strands stops the cancer cells from replicating.

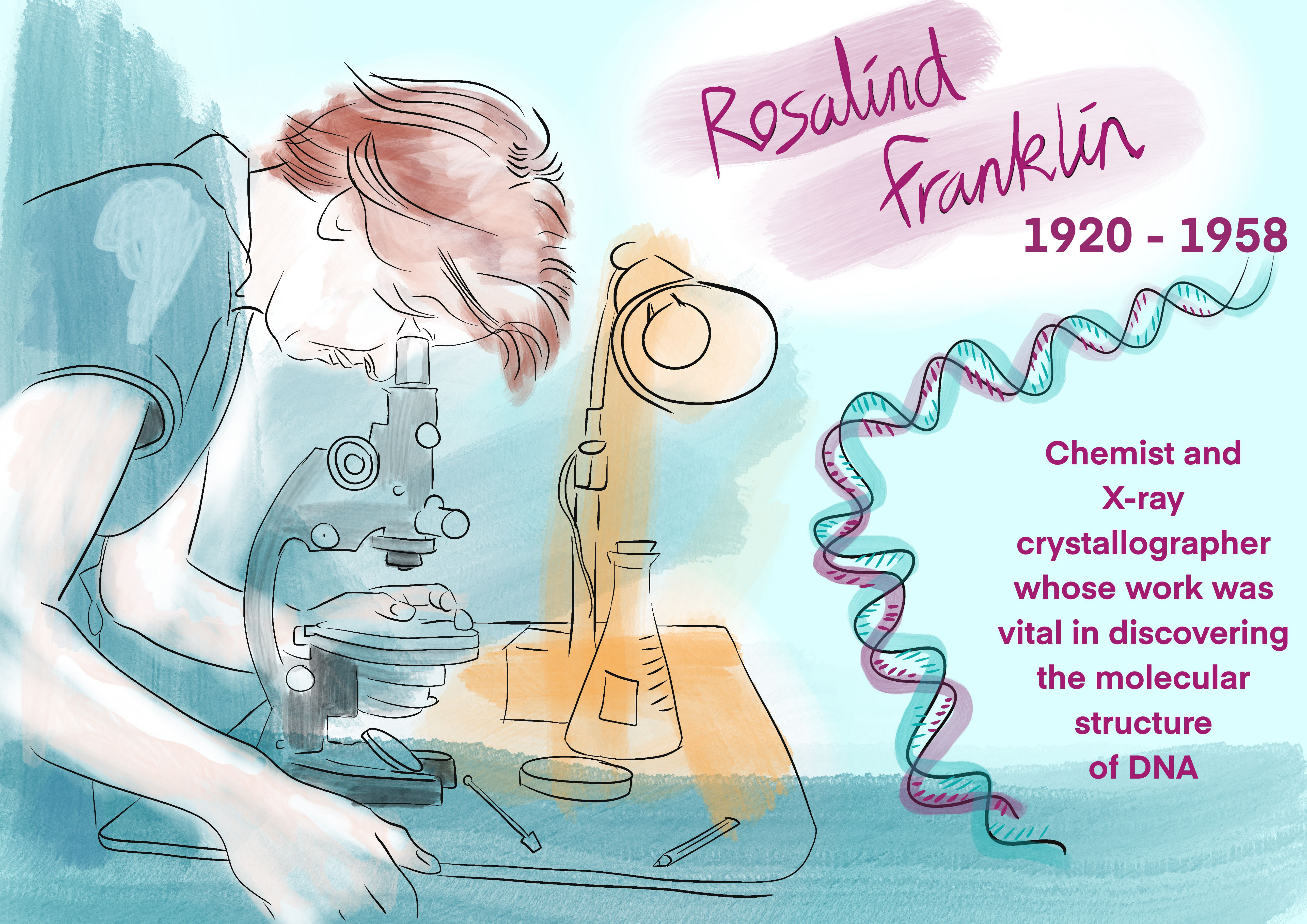
Rosalind Franklin
1920 - 1958
Chemist and X-ray crystallographer whose work was vital in discovering the molecular structure of DNA

Amino acids & proteins

CHEM zine #9

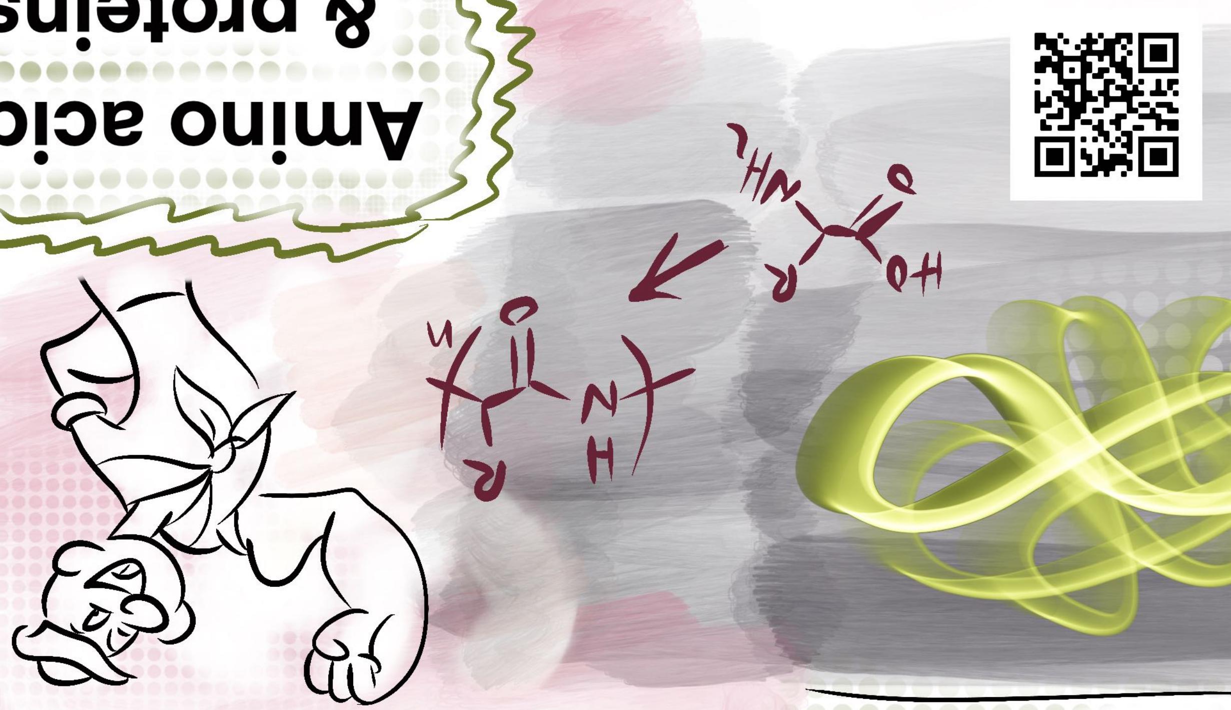

By Alexander Cook

Chemistry learning with comics! More zines, quizzes, and activities at chemzine.com

Copyright © Cook, 2022, CC by 4.0

Proteins

are polymers of amino acid monomers. (20 naturally occurring)

* Make up the majority of cells & living organisms.
* Perform functions inside our bodies as enzymes.

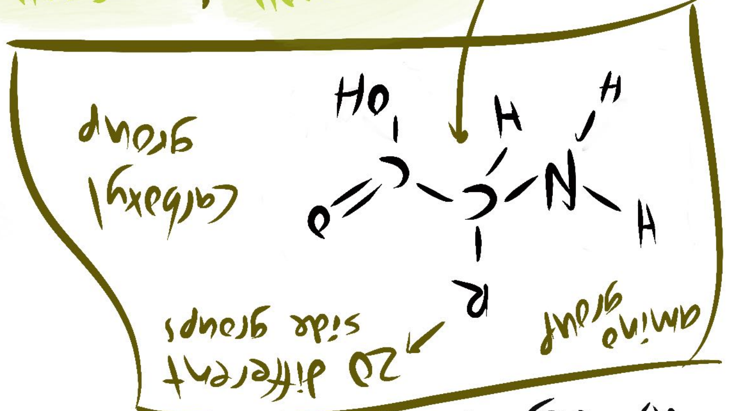

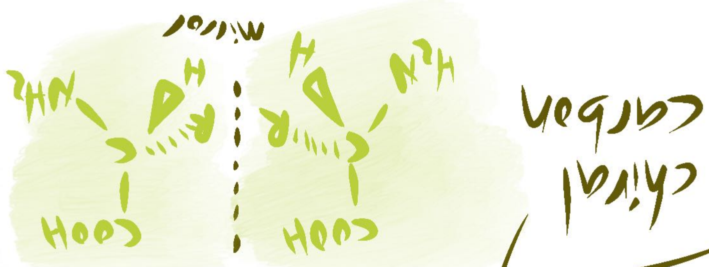

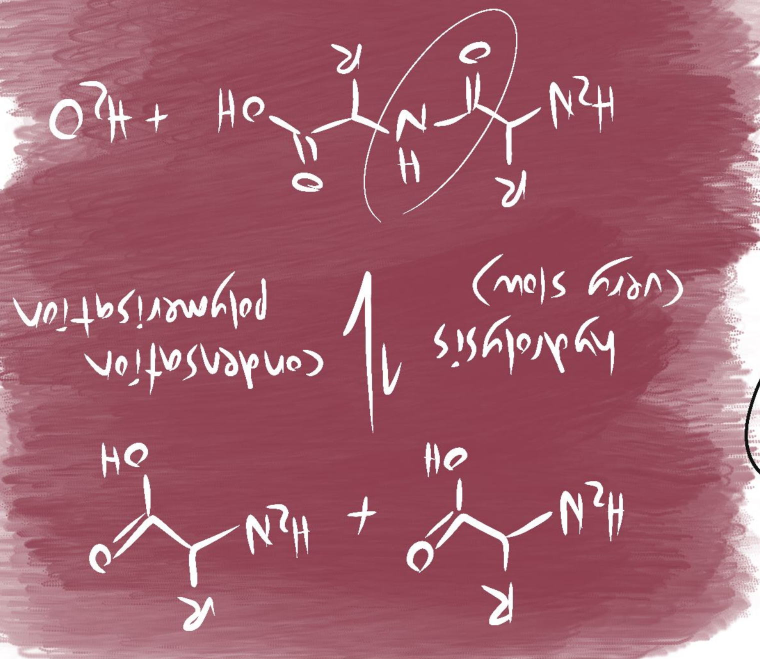

* can undergo further reactions to make longer amino acid chains (or proteins).

Protein structure

Primary structure particular sequence of amino acids

eg HOOC-leucine-Arginine-cysteine-histidine-lysine-glycine-H_2N (C terminus, N terminus)

Secondary structure describes how protein chains are held together by H-bonding between amide bonds

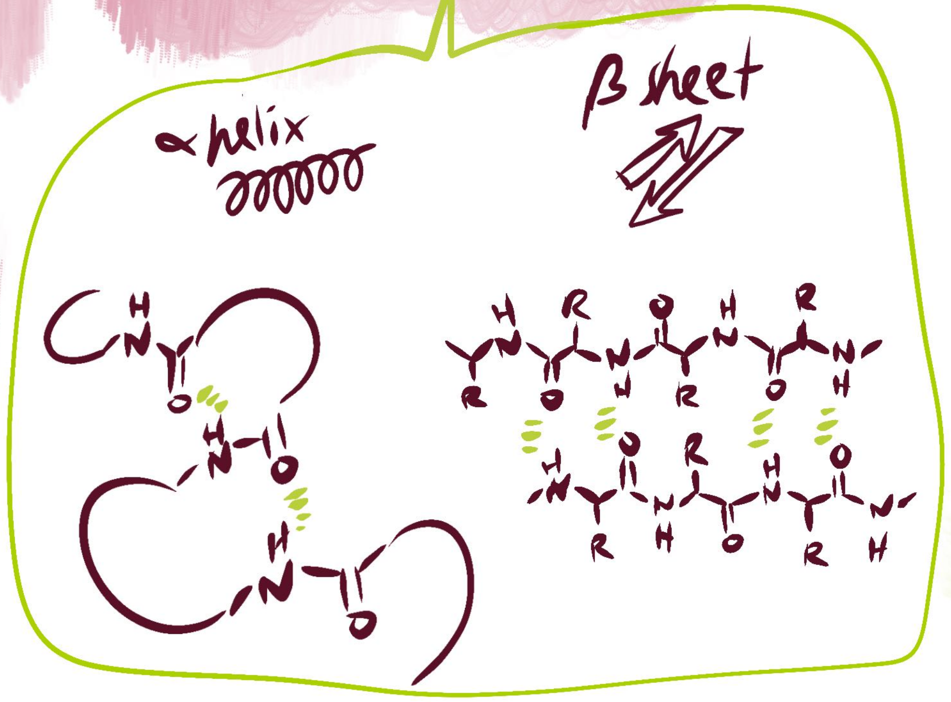

Tertiary structure

additional folding of overall protein due to side chain 'R' group intermolecular interactions ⇒ 3D shape

Enzymes

* type of protein that does a specific job.
* enable particular reactions in the body by acting as a catalyst.

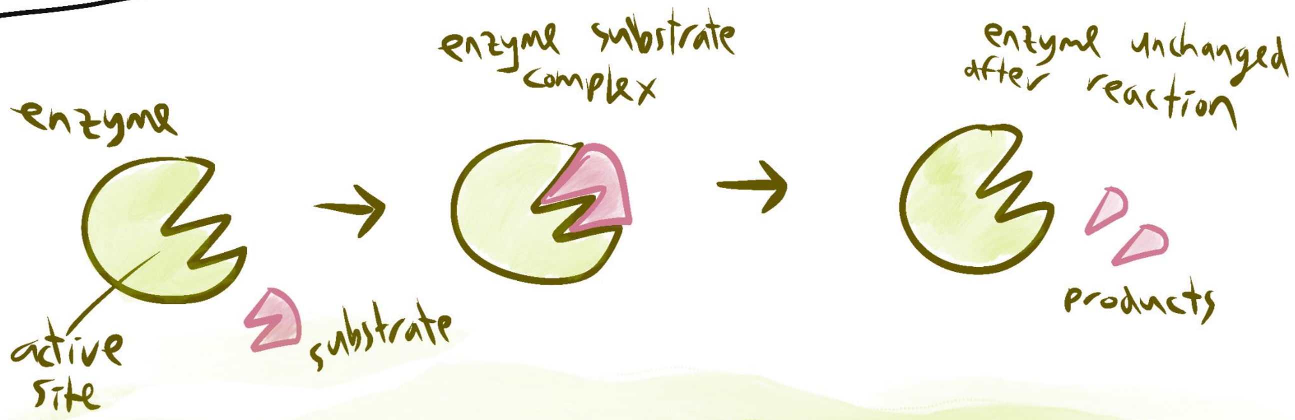

Enzyme inhibitors as drug molecules

if a protein/enzyme is causing a disease, molecules can be designed to bind to the active site as a treatment.

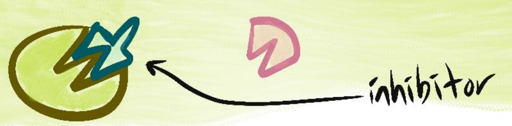

Dorothy Hodgkin

1910 - 1994

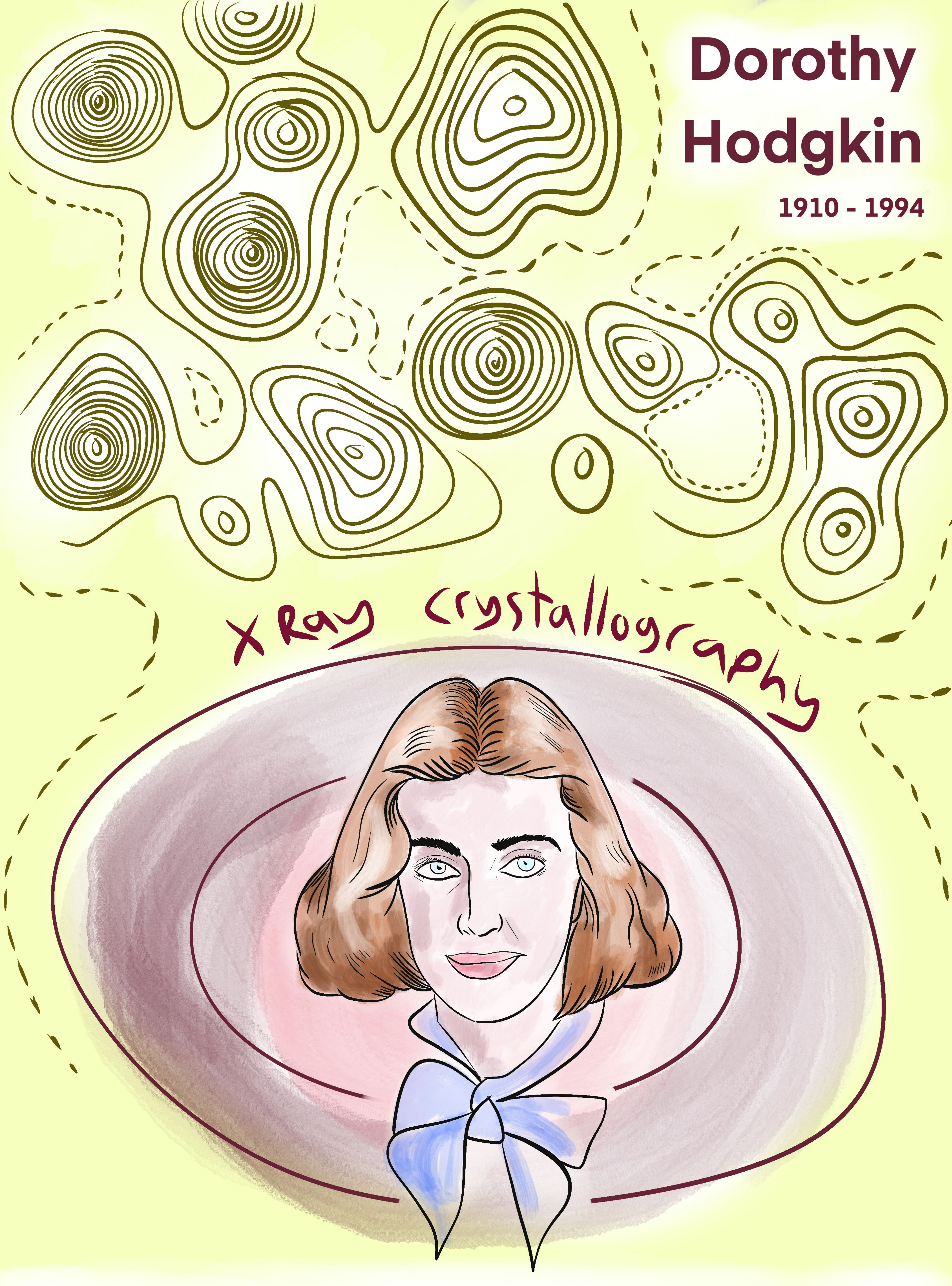

Hodgkin pioneered crystallography techniques to determine protein structure, including insulin in 1969 after 35 years of work.
